# Membrane recruitment of Atg8 by Hfl1 facilitates turnover of vacuolar membrane proteins in yeast cells approaching stationary phase

**DOI:** 10.1186/s12915-021-01048-7

**Published:** 2021-06-04

**Authors:** Cheng-Wen He, Xue-Fei Cui, Shao-Jie Ma, Qin Xu, Yan-Peng Ran, Wei-Zhi Chen, Jun-Xi Mu, Hui Li, Jing Zhu, Qingqiu Gong, Zhiping Xie

**Affiliations:** 1 State Key Laboratory of Microbial Metabolism and Joint International Research Laboratory of Metabolic & Developmental Sciences, School of Life Sciences and Biotechnology, Shanghai Jiao Tong University, Shanghai, 200240 China; 2 Present address: Department of Genetics, Yale School of Medicine, New Haven, CT 06510 USA

**Keywords:** Microautophagy, Vacuole, Yeast, Atg8, Hfl1, ESCRT

## Abstract

**Background:**

The vacuole/lysosome is the final destination of autophagic pathways, but can also itself be degraded in whole or in part by selective macroautophagic or microautophagic processes. Diverse molecular mechanisms are involved in these processes, the characterization of which has lagged behind those of ATG-dependent macroautophagy and ESCRT-dependent endosomal multivesicular body pathways.

**Results:**

Here we show that as yeast cells gradually exhaust available nutrients and approach stationary phase, multiple vacuolar integral membrane proteins with unrelated functions are degraded in the vacuolar lumen. This degradation depends on the ESCRT machinery, but does not strictly require ubiquitination of cargos or trafficking of cargos out of the vacuole. It is also temporally and mechanistically distinct from NPC-dependent microlipophagy. The turnover is facilitated by Atg8, an exception among autophagy proteins, and an Atg8-interacting vacuolar membrane protein, Hfl1. Lack of Atg8 or Hfl1 led to the accumulation of enlarged lumenal membrane structures in the vacuole. We further show that a key function of Hfl1 is the membrane recruitment of Atg8. In the presence of Hfl1, lipidation of Atg8 is not required for efficient cargo turnover. The need for Hfl1 can be partially bypassed by blocking Atg8 delipidation.

**Conclusions:**

Our data reveal a vacuolar membrane protein degradation process with a unique dependence on vacuole-associated Atg8 downstream of ESCRTs, and we identify a specific role of Hfl1, a protein conserved from yeast to plants and animals, in membrane targeting of Atg8.

**Supplementary Information:**

The online version contains supplementary material available at 10.1186/s12915-021-01048-7.

## Background

The vacuole/lysosome is a single-membrane lytic organelle [1]. Its limiting membrane separates the rest of the cytosol from its lumenal hydrolases. To get degraded in the vacuole/lysosome, cellular components need to be delivered to its lumen by one of several autophagic pathways [2–5]. For reasons not fully understood, the limiting membrane of vacuole/lysosome, including proteins embedded therein, is not attacked by its own hydrolases under normal conditions, which would otherwise lead to permeablization of the limiting membrane and possibly cell death [6]. However, both membrane damage and shift of metabolic activity can trigger the elimination of vacuoles/lysosomes by autophagic pathways, either in whole or in part.

In macroautophagy, cargos to be degraded are sequestered into double-membrane autophagosomes. The fusion of autophagosomes with functional lysosomes/vacuoles exposes the inner vesicles for hydrolysis. In the majority of cases, the autophagy-related (Atg) proteins mediate the generation of autophagosomes from cytoplasmic membrane sources. This is also true for lysophagy, the elimination of lysosomes by autophagy [7, 8]. Notably, both dependence on Atg proteins and decoration of damaged lysosomes by LC3-positive membranes provide strong evidence that lysophagy is a form of selective macroautophagy. LC3 belongs to the Atg8 family of ubiquitin-like proteins (UBL). The association of Atg8s with autophagosomal membranes, including those targeting damaged lysosomes, requires their conjugation to phosphatidylethanolamine (PE) [9]. It occurs in a series of reactions catalyzed by several enzymes, including Atg7, Atg3, and a second UBL containing complex, the Atg12-Atg5-Atg16 complex. The conjugation to PE is reversible, and the deconjugation reaction is catalyzed by the Atg4 family of proteases. In the absence of deconjugation, Atg8 accumulates in the membrane-bound form and macroautophagy is defective [10–12]. Like many other selective autophagy pathways, ubiquitination of lysosomal membrane proteins served as an important signal in the initiation of lysophagy [13–15].

In microautophagy, generally, the vacuolar/lysosomal membrane itself is employed for cargo sequestration. Pits are formed by either protrusion or invagination of vacuolar/lysosomal membrane. These pits become lumenal vesicles upon scission of pit necks. In principle, microautophagy is always accompanied by turnover of the membrane constituents of the lumenal vesicles, leading to partial elimination of vacuoles/lysosomes. However, the simultaneous turnover of both cytoplasmic materials and vacuolar/lysosomal membrane components is not examined in most studies. The molecular machineries mediating microautophagy are more diverse than that of macroautopahgy [5]. Depending on the experimental condition, it may rely on many of the Atg proteins to various degrees, ranging from utilizing almost the entire set to being completely independent. A prime example of microautophagy with extensive utilization of Atg proteins (more than twenty of them) is the elimination of peroxisomes in yeast *Pichia pastoris* upon shift of carbon source [16], suggesting that the function of Atg proteins is not limited to the generation of double-membrane vesicles. A subset of microautophagic processes, which are mostly Atg-independent, utilize the endosomal-sorting-complexes-required-for-transport (ESCRT) machinery. The ESCRT proteins were initially discovered in the study of multi-vesicular body (MVB) formation at endosomes [17, 18]. They are generally organized into four complexes (from ESCRT-0 to ESCRT-III), which act to deform membrane and concentrate cargos, and an ATPase complex (Vps4-Vta1) responsible for the disassembly of ESCRT-III filaments. Recent evidence suggests that the ESCRTs are also involved in the generation of lumenal vesicles from the vacuolar membrane, leading to eventual degradation of membrane proteins [19–21]. In particular, ESCRTs mediate the selective turnover of several vacuolar membrane transporters upon alteration of amino acid availability, which is triggered by ubiquitination of the transporters [22]. The ESCRTs are also employed in the repair of damaged lysosomes/endosomes that occurs prior to Atg-mediated lysophagy [23–25]. At present, it is not clear if the repair process leads to elimination of membrane components.

Microlipophagy, the microautophagic turnover of lipid droplets (LD), represents a unique case of mechanistic diversity for a single type of substrate. Despite superficially similar morphological descriptions, some studies reported strong dependence on the core Atg proteins, others instead pointed to the ESCRT proteins as the major players [19, 26–28]. For microlipophagy, membrane invaginations appear to occur at sterol-enriched membrane microdomains [27, 29]. Both the Atg machinery and the ESCRT machinery have been implicated in the formation of membrane microdomains. The vacuolar localization of the Niemann-Pick type C (NPC) proteins is regulated by the Atg machinery [29]. Thus, the role of Atg proteins in microlipophagy may in part be attributed to the NPC proteins, which participate in microdomain expansion by supplying sterols to the vacuolar membrane.

Here, we present evidence that in *Saccharomyces cerevisiae* cells approaching stationary phase, vacuolar membrane proteins are degraded in the lumen of this compartment. It is mechanistically distinct from most known autophagic pathways.

## Results

### Degradation of vacuolar membrane proteins in early stationary phase

**Fig. 1:**
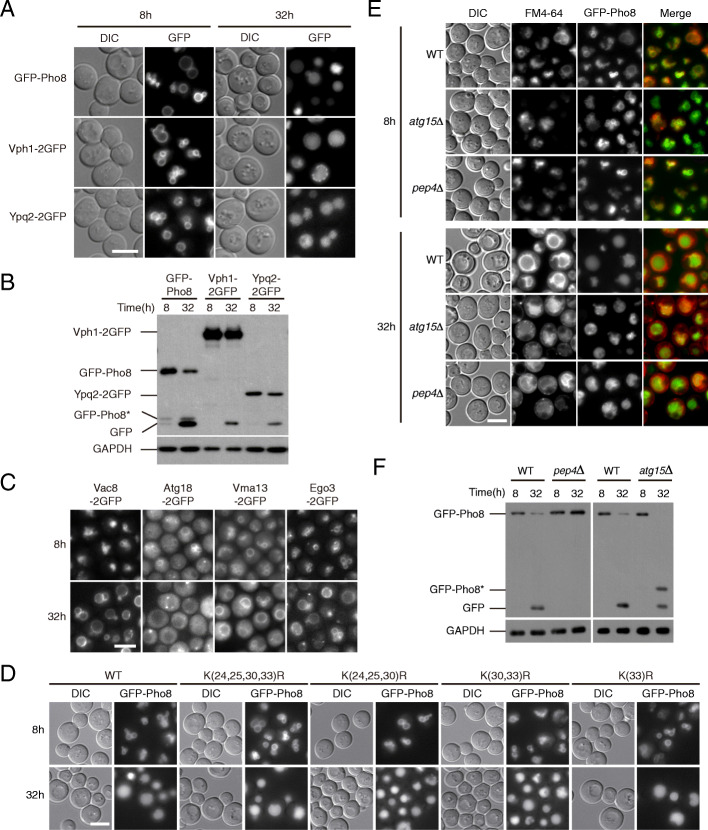
Vacuolar membrane proteins are degraded in cells approaching stationary phase. **a**, **b** Integral vacuolar membrane proteins with diverse functions are degraded in cells approaching stationary phase. At time 0, log phase yeast cultures expressing the indicated GFP fusion proteins were diluted to OD_600_=0.2. Images and samples were collected at the indicated time points afterwards. **a** Fluorescent microscopy images showing the translocation of GFP signal from the surface to the lumen of vacuoles. DIC, differential interference contrast. Scale bar, 5 μm. **b** Immuno-blots showing the formation of free GFP bands. GFP-Pho8* presumably represents a truncated form. **c** Peripheral vacuolar membrane proteins do not translocate to vacuolar lumen in early stationary phase. Cells treated and images presented as in **a**. **d** Lysines at the cytosolic domain of Pho8 are dispensable for its turnover. The *PHO8* locus was replaced with *pho8* mutants carrying lysine-arginine substitutions as indicated. Cells treated and images presented as in **a**. **e**, **f** Turnover of GFP-Pho8 is dependent on vacuolar hydrolases. Cells were treated as in **a**. **e** Fluorescent microscopy images showing the appearance of uneven GFP signal in vacuoles in *pep4Δ* and *atg15Δ* cells. FM4-64 was used to label the vacuolar membrane. **f** Immuno-blots showing defective formation of free GFP in *pep4Δ* and *atg15Δ* cells

To investigate potential pathways mediating the turnover of the vacuolar membrane, we tagged multiple integral membrane proteins functioning in different processes with green fluorescent protein (GFP), and searched for conditions that could result in their translocation into the vacuolar lumen. Among proteins reported to be on the vacuole membrane, we picked proteins that exclusively displayed a vacuolar rim pattern in log-phase (Dpp1, Ncr1, Pho8, Vba4, Vph1, Ycf1, Ypq1, Ypg2, and Ypl162c) and ignored ones that were detectable at other locations. We found that except for Ycf1, substantial GFP signal appeared in the vacuolar lumen when cells gradually exhausted nutrients and approached stationary phase (Fig. 1a, Additional file 1: Fig. S1A) [30]. Immunoblot analysis revealed the presence of free GFP bands, implying that these proteins are subject to degradation (Fig. 1b, Additional file 1: Fig. S1B). We also checked four peripheral membrane proteins with vacuolar association, but did not observe substantial vacuole entry or GFP cleavage (Fig. 1c, Additional file 1: Fig. S1B). These results indicate that a potentially general degradation process occurs for vacuolar integral membrane proteins when cells are entering stationary phase. For convenience, we refer to this phenomenon as “*e*arly-stationary *v*acuole *t*urnover (EVT)” hereafter.

The remaining study was primarily focused on Pho8, as its turnover was the strongest among the examined proteins. By analogy with other membrane proteins [31], we mutated the lysine residues in the cytosolic domain of Pho8 to examine if the turnover depends on its own ubiquitination [32]. Even with all four lysines substituted with arginine, the turnover of Pho8 proceeded normally (Fig. 1d, Additional file 1: Fig. S1C), indicating that direct ubiquitination is not required for this particular protein. It is possible, however, that ubiquitination may be involved in other steps or the targeting of other cargos. To test if the degradation was an artifact resulting from non-functional protein fusion, we measured the alkaline phosphatase activity in *pho8Δ* cells expressing GFP-Pho8 and found it comparable with that of wild-type cells (Additional file 1: Fig. S1D). In addition, translocation and turnover of Pho8 chimeras also occurred with a mRuby3 tag (a red fluorescent protein unrelated to GFP) and a 4myc tag, but not a 4 V5 tag (Additional file 1: Fig. S1E-F), demonstrating that the turnover is not limited to GFP fusions.

**Fig. 2:**
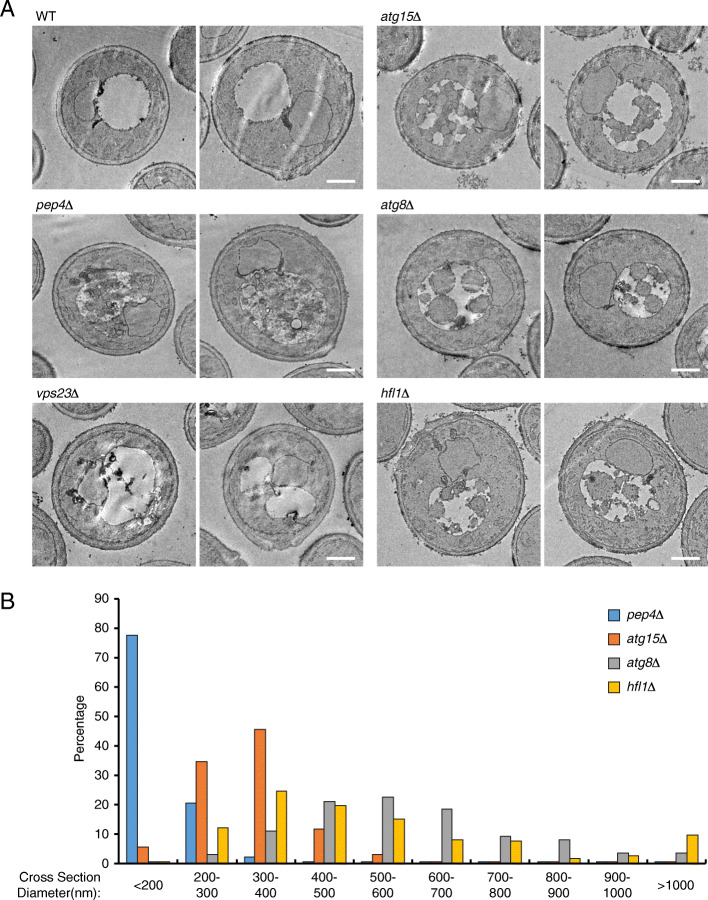
Characterization of EVT by transmission electron microscopy. **a**, **b** Cells of the indicated genotypes were treated as in Fig. 1a. At 32 h after dilution to OD_600_=0.2, cells were collected and processed for TEM. Multiple vesicles accumulated in the vacuoles of *pep4Δ*, *atg15Δ*, *atg8Δ*, and *hfl1Δ* cells. The lumenal vesicles in *atg8Δ* and *hfl1Δ* cells were generally larger than those in *pep4Δ* and *atg15Δ* cells. For each strain, two representative cells are presented (**a**). Scale bar, 1 μm. For each strain that contained a substantial amount of lumenal structures, the diameters of approximately 200 vesicle-like structures were measured. The resulting distributions are presented in **b**

We further examined whether the turnover of Pho8 depends on vacuolar hydrolases and whether some form of vesicular intermediates is involved. In *pep4Δ* and *atg15Δ* cells, we observed uneven distribution of vacuolar GFP signal (Fig. 1e). Transmission electron microscopy (TEM) revealed that the vacuoles of *pep4Δ* and *atg15Δ* cells accumulated multiple vesicles in early stationary phase (Fig. 2a), but not in log phase (data not shown). The vacuoles in *pep4Δ* cells were often filled with substances having similar electron density as the cytosol, making it difficult to recognize the vesicles, many of which were quite small and mixed within the surrounding substances. The vesicles in *atg15Δ* cells were visually larger and easier to recognize, with diameters mostly in the 200–400 nm range (Fig. 2b). Immunoblotting revealed that the formation of free GFP was completely blocked in *pep4Δ* cells and partially blocked in *atg15Δ* cells (Fig. 1f). The difference in the severity of the phenotypes was consistent with the paramount role of Pep4 in the activation of vacuolar hydrolases and a more limited role of Atg15 as a lipase [33–35]. In *atg15Δ* cells, active proteases presumably had access to the lumenal domain of Pho8, resulting in the accumulation of a truncated Pho8 variant (Pho8*) that had potentially shed its lumenal domain [36]. The Pho8* variant was absent in *pep4Δ* cells and only occasionally detected in wild-type cells (see also Fig. 1b). These data indicate that EVT is a process that degrades self-constituents (vacuolar proteins) in the vacuole, utilizing vacuolar hydrolases, which fits the classical concept of autophagy.

It is worth noting that when we used the usual approach of labeling the vacuole with FM4-64 (Fig. 1e), we found that the transport of this lipophilic dye from the plasma membrane (PM) to the vacuolar membrane in early stationary phase was substantially slower than in log phase. The process in *pep4Δ* and *atg15Δ* cells was even slower, with many cells still displaying PM and punctate signal 3 h after the dye was flushed out. At present, the significance of this observation is not understood.

### EVT depends on a macroautophagy-independent function of Atg8

**Fig. 3:**
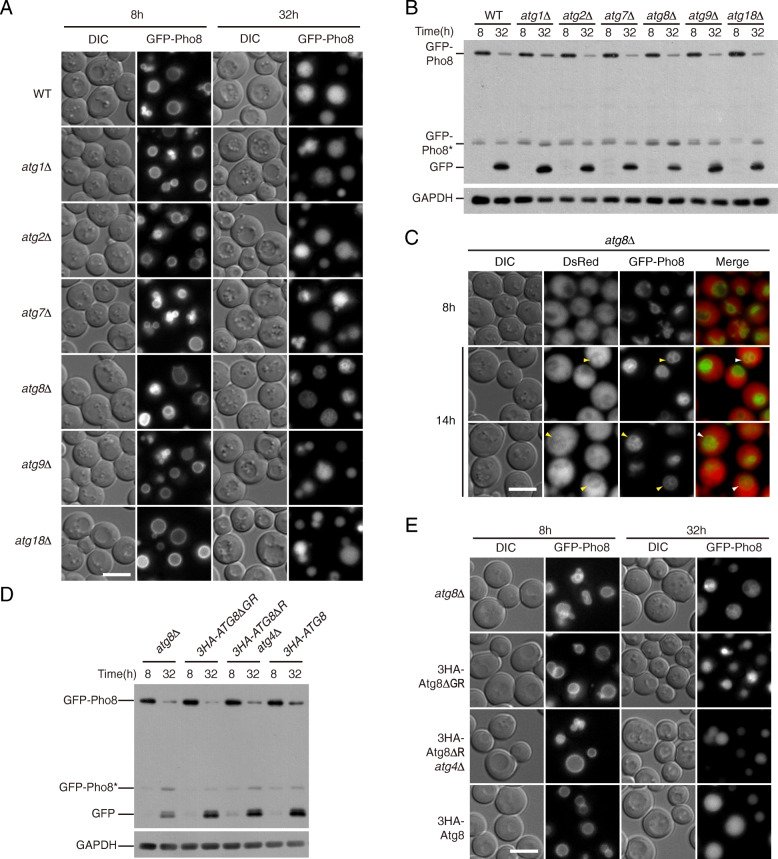
EVT involves a conjugation-independent function of Atg8. **a**, **b** The degradation of GFP-Pho8 is independent of most *ATG* genes except for *ATG8*. Cells were treated as Fig. 1a. Data are presented as in Fig. 1a and b, respectively. **a** *atg8Δ* cells accumulated vesicle-like structures within vacuoles. **b** *atg8Δ* cells contained less free GFP and accumulated a truncated form of Pho8. See Fig. S2A for quantification. **c** Cytosol is sequestered in the intravacuolar structures in *atg8Δ* cells. *atg8Δ* cells co-expression GFP-Pho8 and DsRed were imaged at the indicated time points after being diluted to OD_600_=0.2. Arrowheads, DsRed signal contained within GFP-Pho8 demarcated zones inside vacuoles. **d**, **e** Atg8-PE conjugation and deconjugation are dispensable in EVT. GFP-Pho8 expressing cells with the indicated genotype and Atg8 variants were treated as in Fig. 1a. **d** Fluorescent microscopy images assessing the accumulation of intravacuolar structures. **e** Immuno-blots assessing the proteolytic processing of GFP-Pho8. See Fig. S2C for quantification

Next, we examined how the EVT phenomenon relates to known autophagic pathways in yeast. Macroautophagy (either selective or non-selective) relies on the core autophagy machinery. As macroautophagy-like processes have been reported in other species that operate in the absence of certain core machinery genes [37, 38], we tested several knockout strains that lack Atg proteins in different functional groups [2]. In *atg1Δ*, *atg2Δ*, *atg7Δ*, *atg9Δ*, and *atg18Δ* cells, both the translocation of GFP-Pho8 into the vacuole lumen and the processing into free GFP occurred normally (Fig. 3a, b; Additional file 1: Fig. S2A), indicating that EVT is overall independent from macroautophagy. In contrast, *atg8Δ* cells accumulated internal GFP-positive structures resembling vesicles (Fig. 3a). The unique morphology of *atg8Δ* cells was accompanied by a lower level of free GFP and a higher level of GFP-Pho8* (Fig. 3b, Additional file 1: S2A). The increase in GFP-Pho8* level implies that these Pho8 molecules were trapped on membranes longer than normal, like in *atg15Δ* cells. Similar results were obtained using Vph1-2GFP (Additional file 1: Fig. S2B), indicating that the unique role of Atg8 is a general feature of EVT. In TEM, the intravacuolar structures in *atg8Δ* cells were visibly larger than those in *pep4Δ* and *atg15Δ* cells, reaching up to 1 um in diameter (Fig. 2a, b). Among these structures, around 5% appeared to be membrane invaginations with clear openings towards the cytosol, and 83% were vesicle-like with boundaries clearly separate from the vacuolar membrane, with the rest being difficult to tell. In cells co-expressing a cytosolic RFP protein (DsRed), red fluorescent signal was detectable inside these Pho8-demarcated structures (Fig. 3c), indicating that their formation is accompanied by sequestration of cytosol. As controls, we confirmed that all *atg* knockout strains, including *atg8Δ*, were defective in macroautophagy (data not shown).

The function of Atg8 in macroautophagy depends on its reversible conjugation with PE [39]. Consistent with the fact that Atg7 (the E1-like enzyme) is dispensable (Fig. 3a, b), the translocation and turnover of Pho8 occurred normally in *atg8Δ* cells expressing Atg8ΔGR (a non-conjugatable form) (Fig. 3d, e; Additional file 1: S2C-D). EVT was also normal in *atg8Δ atg4Δ* cells expressing Atg8ΔR, which accumulated membrane-bound Atg8-PE. These data suggest that the way that Atg8 participates in EVT is different from that in macroautophagy.

### EVT is distinct from several other known autophagic pathways

We further tested the potential involvement of genes functioning in the piecemeal microautophagy of the nucleus (PMN) pathway and the vacuole import and degradation (VID) pathway. PMN is a microautophagic process that eliminates small portions of the nuclear envelope [40]. In addition to the core Atg proteins, PMN requires Vac8 [41]. VID mediates the turnover of several metabolic enzymes functioning in gluconeogenesis [42–44]. It depends on the *VID* genes, but not *ATG* genes. We found no substantial defects in the translocation and turnover of GFP-Pho8 in *vac8Δ*, *vid24Δ*, or *vid28Δ* cells (Additional file 1: Fig. S3A-B, S3D-E). As controls, we verified that degradation of Osh1 (a PMN substrate) and Fbp1 (a VID substrate) was blocked in the mutants of the corresponding pathways (Additional file 1: Fig. S3C, S3F). We also tested two protein complexes reported to participate in microautophagy, the VTC complex and the EGO complex [45, 46]. Under our experimental condition, there was no obvious defect of Pho8 turnover in *vtc1Δ*, *vtc2Δ*, *vtc3Δ*, *vtc4Δ*, *ego1Δ*, *gtr2Δ*, or *ego3Δ* cells (Additional file 1: Fig. S4A-D).

Under certain conditions, microautophagy is employed to degrade LDs. It has been suggested that LD sequestration relies on the formation of lipid microdomains, which involves the supply of sterols by the NPC proteins. As cells approached stationary phase, LDs could eventually be detected in the vacuole lumen (Additional file 1: Fig. S5A), albeit the timing was much later than the internalization of Pho8. Furthermore, *ncr1Δ npc2Δ* cells displayed defective LD sequestration, but normal GFP-Pho8 internalization (Additional file 1: Fig. S5A-B). These data suggest that EVT is different from these reported microlipophagy processes.

### Atg8 functions downstream of the ESCRT machinery

**Fig. 4:**
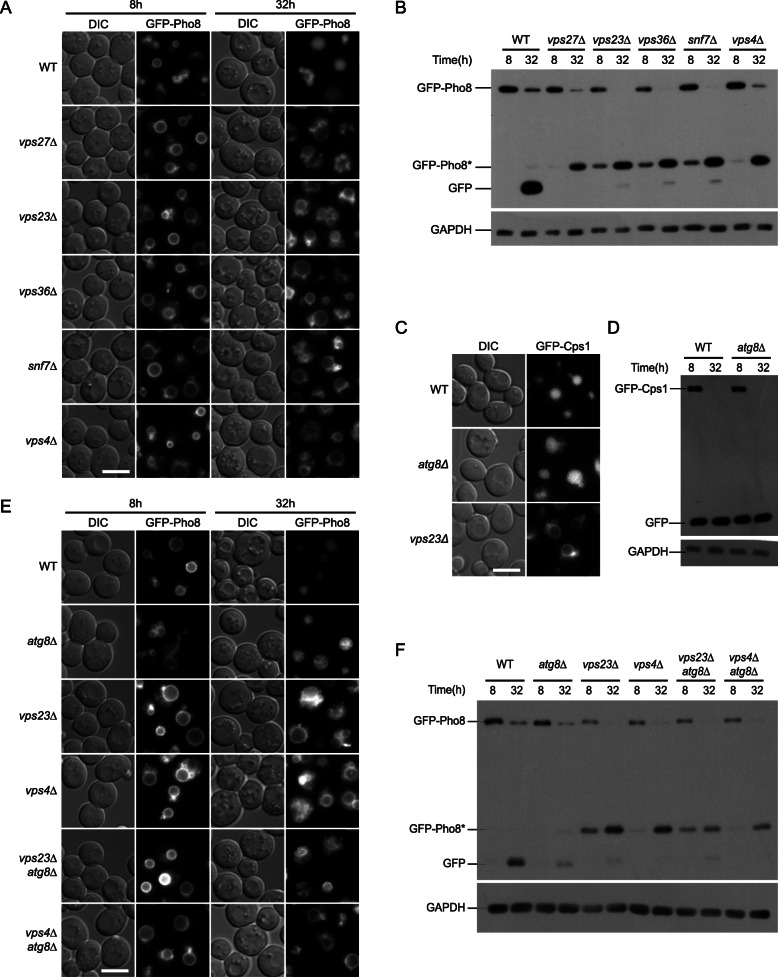
Atg8 acts downstream of ESCRT proteins in EVT. **a**, **b** The degradation of GFP-Pho8 is defective in ESCRT mutants. Cells were treated as Fig. 1a. Data are presented as in Fig. 1a and b, respectively. **c**, **d** The MVB pathway does not depend on Atg8. **c** Sorting of GFP-Cps1 monitored by fluorescent microscopy. Mid-log phase yeast cells were collected. **d** Processing of GFP-Cps1 into free GFP monitored by immuno-blot. **e**, **f** Epistatic analysis of *atg8Δ* and ESCRT mutants. GFP-Pho8 expressing cells with the indicated genotypes were treated as Fig. 1a. Data are presented as in Fig. 1a and b, respectively. See Fig. S6B for quantification of immune-blots

We then examined the role of the ESCRT proteins and found that *vps27Δ* (ESCRT-0), *vps23Δ* (ESCRT-I), *vps36Δ* (ESCRT-II), *snf7Δ* (ESCRT-III), and *vps4Δ* mutants failed to deliver GFP-Pho8 into the vacuole in early stationary phase (Fig. 4a). Immunoblot analysis indicates that instead of producing free GFP, these cells almost exclusively accumulated the partially truncated Pho8* (Fig. 4b). The translocation of Vph1-2GFP was also defective in these mutants (Additional file 1: Fig. S6A). The dependence on all the ESCRT complexes implies that EVT is mechanistically related to MVB formation at endosomes. However, although all ESCRT mutants accumulated Cps1 (a MVB pathway cargo) at the “class E” compartment [47], the localization of and processing of GFP-Cps1 appeared normal in *atg8Δ* cells (Fig. 4c, d). This is consistent with the absence of *atg8* in the initial *vps* mutant screen and indicates that internalization of vacuolar membranes involves unique mechanisms.

To understand the epistatic relationship between Atg8 and ESCRTs, we constructed *vps23Δ atg8Δ* and *vps4Δ atg8Δ* double mutants. Unlike *atg8Δ* cells, these double knockout cells maintained GFP signal on the vacuolar membrane and did not accumulate GFP-positive structures in the vacuole (Fig. 4e). Immuno-blot analysis indicated that the double mutants primarily contained the truncated form of GFP-Pho8*, similar to single *vps* mutants (Fig. 4f, Additional file 1: S6B). These results suggest that Atg8 acts downstream of the ESCRT proteins in EVT.

### The internalization step of EVT occurs on the vacuole

**Fig. 5:**
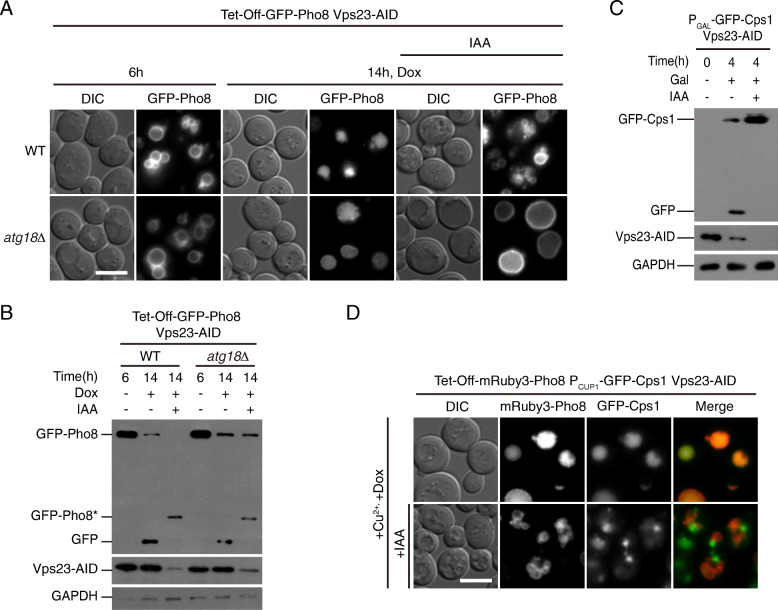
The internalization step of EVT occurs on the vacuole. **a**, **b** Depletion of Vps23 prevents the turnover of pre-existing Pho8. In these cells, expression of GFP-Pho8 was controlled by the tet-off promoter, and Vps23 was tagged with the AID degron. Six hours after dilution to OD_600_=0.2, chemicals to shut-off Pho8 expression (1 μg/ml doxycycline) (Dox) or deplete Vps23 (500 uM IAA) were added as indicated. Cells were then incubated for another 8 h. **a** Translocation of GFP signal monitored by fluorescent microscopy. Scale bar, 5 μm. **b** Turnover of GFP-Pho8 and Vps23-AID monitored by immuno-blot. **c** Control experiment showing that depletion of Vps23 prevents vacuolar sorting of newly synthesized Cps1. Cells expressing GFP-Cps1 under the control of *GAL1* promoter and Vps23 tagged with AID were cultured as in A, except for using a raffinose-containing medium (SMR+CA). Cells were then treated with or without IAA for 2.5 h. Finally, galactose was added to induce Cps1 expression for 1 h. Processing of GFP-Cps1 was monitored by immuno-blot. **d** Depletion of Vps23 does not cause the relocation of pre-existing Pho8 to endosomes. Cells expressing mRubby3-Pho8 under the control of the tet-off promoter, GFP-Cps1 under the *CUP1* promoter and Vps23 tagged with AID were treated as in **a**, except that copper sulfate was added together with doxycycline. After 8 h, cells were imaged by fluorescent microscopy. Scale bar, 5 μm

The close proximity between endosomes and the vacuole makes it difficult to observe by fluorescent microscopy if ESCRTs act directly on the vacuolar membrane. We therefore used a pulse-chase assay to test the effect of transient ESCRT protein depletion on pre-existing Pho8. Shutting off the expression of GFP-Pho8 at log phase using the tet-off system did not prevent the appearance of lumenal GFP signal and the processed free GFP band (Fig. 5a), confirming that pre-existing Pho8 on the vacuole is subject to translocation. When Vps23 was provisionally depleted using the auxin-inducible degron (AID) system [48], the generation of free GFP was blocked (Fig. 5b). The GFP signal on the surface of vacuoles became somewhat trickier to see under this condition, as the depletion of Vps23 augmented the fragmentation of vacuoles. To overcome this technical issue, we blocked a vacuole-to-Golgi trafficking pathway by knocking out *ATG18* to make the vacuoles larger [49]. In those cells, the GFP signal remained on the vacuolar membrane when Vps23 was depleted (Fig. 5a). We further verified that upon depletion of Vps23, newly synthesized GFP-Cps1 (expressed by an inducible *CUP1* promoter) accumulated on punctate structures resembling typical “class E compartment” and failed to get proteolytically processed (Fig. 5c, d). But these Cps1 positive structures did not contain Pho8 (Fig. 5d). Note that for these set of experiments, we picked a time point earlier than previous ones (14 h instead of 32 h) in order to observe the immediate impacts of depleting relevant proteins. At this stage, the degradation was already detectable (Additional file 1: Fig. S6C). Overall, these data indicate that in EVT, Pho8 is internalized at the vacuole, without transit through endosomes.

### Hfl1 recruits Atg8 to vacuolar membrane to facilitate EVT

**Fig. 6:**
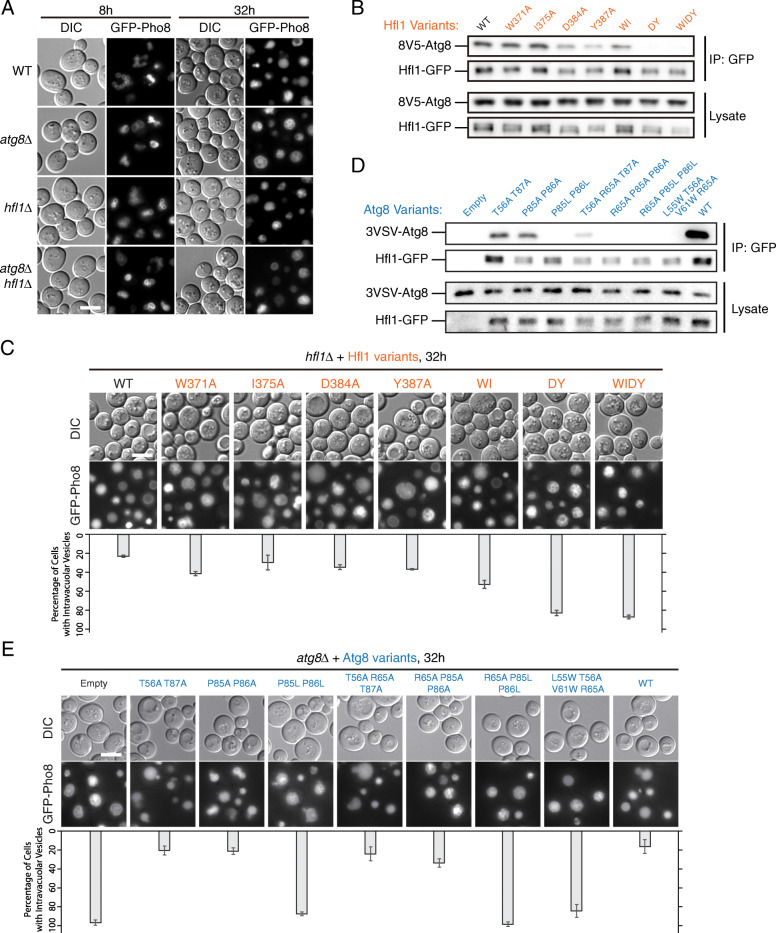
Hfl1 and Atg8 function at the same step in EVT. **a** *hfl1Δ* cells and *atg8Δ hfl1Δ* cells accumulate intravacuolar structures similar to *atg8Δ* cells. Cells treated and image presented as in Fig. 1a. **b**, **c** *hfl1* mutants defective in Hfl1-Atg8 interaction accumulate intravacuolar structures. WI, W371A I375A; DY, D384A Y387A; WIDY, W371A I375A D384A Y387A. **b** Assessment of Hfl1-Atg8 interaction by co-immunoprecipitation. In these cells, Hfl1-GFP was expressed under the *TEF1* promoter, and 8 V5-Atg8 under its own promoter. Cells in log phase were collected. **c** Observation of GFP-Pho8 translocation by fluorescent microscopy. Cells treated as in Fig. 1a. Representative images are presented on top. For each strain, at least 160 cells from three independent repeats were analyzed for the presence of intravacuolar structures. The results are presented below the microscopy images. Error bar, standard deviation, *n*=3. **d**, **e** *atg8* mutants defective in Hfl1-Atg8 interaction accumulate intravacuolar structures. **d** Identification of *atg8* mutants deficient in Hfl1 interaction. Hfl1-Atg8 interaction was assessed by co-immunoprecipitation as in **b**. **e** Observation of GFP-Pho8 translocation by fluorescent microscopy. Cells treated and data presented as in **c**

As the lipidation of Atg8 is not required for EVT, one would expect that an alternative mechanism is utilized for the membrane recruitment of Atg8. Hfl1, an Atg8-interacting vacuolar membrane protein, has been shown to work with Atg8 in the formation of lipid microdomains [50]. Although our data indicate that microdomain *per se* is not essential for EVT (Additional file 1: Fig. S5B), we found that *hfl1Δ* cells accumulated the same type of lumenal structures as *atg8Δ* cells (Figs. 2a, b and 6a; Additional file 1: Fig. S7B-C). The phenotype of the *hfl1Δ atg8Δ* double mutants was comparable with the single mutants (Fig. 6a, Additional file 1: Fig. S7A), implying that Hfl1 and Atg8 act at the same stage in EVT that dictate the appearance of intravacuolar membranes.

We then tested whether the interaction between Hfl1 and Atg8 is important for EVT. Hfl1 contains seven transmembrane domains and interacts with Atg8 using its C-terminal cytosolic region (amino acid residues 368–389) [50]. In line with the finding by Liu et al., we detected severe disruptions of Hfl1-Atg8 interaction by the W371A I375A D384A Y387A (WIDY) quadruple mutation in Hfl1 (Fig. 6b). But the contributions of individual residues to the interaction of whole proteins appeared to differ from in vitro results using the C-terminal peptide of Hfl1. We found that D384A and Y387A mutations that respectively target salt bridges with R65 of Atg8 and the hydrophobic interactions at the Y site pocket each produced a noticeable drop in co-immunoprecipitation (co-IP) efficiency. When combined, the D384A Y387A (DY) double mutation led to a level of interaction disruption close to that of WIDY quadruple mutation. In contrast, mutating W371 alone or in combination with I375 did not substantially decrease Hfl1-Atg8 co-IP efficiency. The strength of the interaction correlated with the resulting in vivo phenotype, with WIDY and DY mutants of Hfl1 producing the strongest accumulation of lumenal GFP-Pho8 structures, and the rest of the point mutants displaying milder phenotypes (Fig. 6c).

To better understand the interaction mechanism, we then studied the interaction interface on Atg8. Consistent with interactions close to the Y pocket being important for the whole proteins, we found that several mutation combinations targeting this region severely reduced Hfl1-Atg8 co-IP efficiency, including P85L P86L, R65A P85L P86L, and L55W T56A V61W R65A (Fig. 6d). Presumably, the introduction of large side chains by P85L, P86L, and V61W altered the shape of the Y pocket that would otherwise accommodate the Hfl1 Y387 side chain (Additional file 1: Fig. S7D). In comparison, without large side chains, P85A P86A double substitution was insufficient to disengage Hfl1 from Atg8. Besides the Y pocket, the L55W T56A V61W R65A quadruple mutation additionally targets the L pocket and several hydrophilic interactions involving E383 and D384 of Hfl1. Furthermore, we noticed that substitution combinations targeting hydrophilic interactions alone were not very effective. Weak Hfl1-Atg8 interaction remained present with T56A R65A T87A triple substitution that targets several hydrophilic interactions involving E383 and D384 of Hfl1. Finally, for the *atg8* mutants discussed above, their impact on EVT correlated well with the degree of interaction disruption (Fig. 6e). P85L P86L, R65A P85L P86L, and L55W T56A V61W R65A mutants all accumulated lumenal GFP-Pho8 structures to levels similar to *atg8Δ*. The phenotype of mutants that retained some interactions (T56A T87A, P85A P86A, and T56A R65A T87A) was generally closer to that of wild-type cells. The only outlier was the T65A P85A P86A triple substitution, which produced a weak phenotype, possibly reflecting differences between our co-IP condition and the actual cytoplasm. Interestingly, mutations near Y pocket of Atg8 also disrupted macroautophagy, as measured by the pho8Δ60 assay (Additional file 1: Fig. S7E). The amounts of Atg8-PE were substantially reduced by the P85L P86L double substitution (Additional file 1: Fig. S7F). Therefore it appears that the Y pocket of Atg8 is important for both EVT and macroautophagy. To examine the possibility that these mutations resulted in pleotropic structural disruption, we evaluated the stability of Atg8 mutant proteins in vitro by differential scanning calorimetry (DSC). The melting temperatures (Tm) of both P85A P86A (47.1 ± 0.2 °C) and P85L P86L (47.2 ± 0.2 °C) mutants were lower than that of wild-type Atg8 (50.9 ± 0.3 °C) (Additional file 1: Fig. S7G), demonstrating that it was technically difficult to disrupt binding to Hfl1 without affecting the thermostability of Atg8. However, because the Tm of both P85A P86A and P85L P86L mutants were lowered similarly, these data also suggest that reduced thermostability alone cannot account for their phenotypic differences. Overall, our data from both *hfl1* mutants and *atg8* mutants demonstrate that interactions at or near the Y site are critical for the association of Hfl1 with Atg8, and that elimination of these interactions was sufficient to dissociate Atg8 from Hfl1 and impede EVT.

**Fig. 7:**
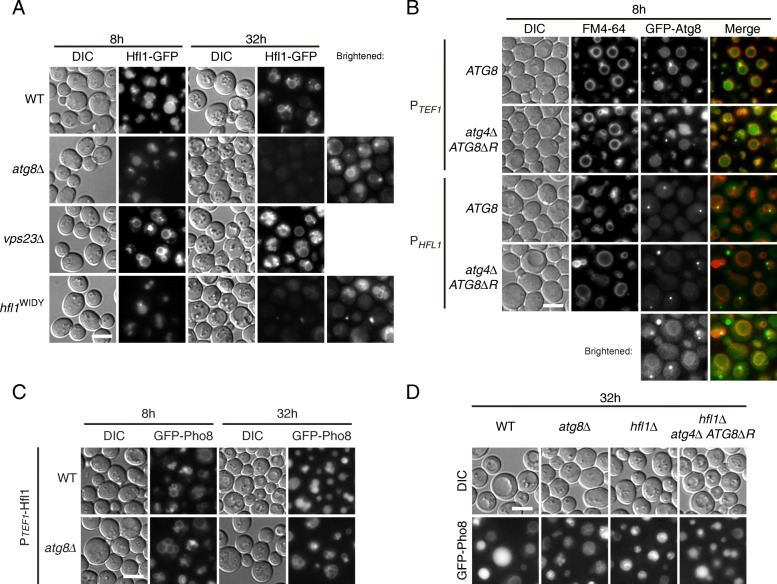
Hfl1 recruits Atg8 to vacuolar membrane to facilitate EVT. **a** The localization of Hfl1 on vacuolar membrane is regulated by its interaction with Atg8. Hfl1-GFP was expressed under the *TEF1* promoter. In the absence of Atg8, or in the case of a mutant that loses the interaction (WIDY), Hfl1 was present in the vacuolar lumen in log-phase, with protein levels reduced in early stationary phase. Cells treated and image presented as in Fig. 1a. **b** Hfl1 promotes the association of Atg8 with vacuolar membrane. Both overexpression of Hfl1 and block of Atg8 delipidation enhanced the signal of GFP-Atg8 on the vacuolar membrane. Cells treated and image presented as in Fig. 1e. **c** Overexpression of Hfl1 does not bypass *atg8Δ*. Hfl1 was overexpressed using *TEF1* promoter in WT and *atg8Δ* cells. Translocation of GFP-Pho8 was assessed as in Fig. 1a. **d** Forced lipidation of Atg8 bypass the need of Hfl1. Translocation of GFP-Pho8 was assessed as in Fig. 1a

Lastly, we investigated the relationship between Hfl1 and Atg8. The expression of Hfl1 at the endogenous level was too low for live-cell imaging of its subcellular localization. When overexpressed by the *TEF1* promoter, the presence of Hfl1-GFP on the vacuolar membrane became clear in both log-phase and early stationary phase (Fig. 7a). Hfl1-GFP remained on the vacuolar surface when *VPS23* was deleted. However, in the absence of Atg8, or in the case of a mutant (WIDY) that lost interaction with Atg8, fluorescence signal was present in vacuolar lumenal in log phase, and became dimmer in early stationary phase, which implies that Atg8 may have a role in maintaining Hfl1 on the vacuolar surface. Possibly resulting from the scarcity of endogenous Hfl1, only when we overexpressed Hfl1 could we distinguish GFP-Atg8 signal on the vacuolar membrane from the cytosolic background during log-phase (Fig. 7b, Additional file 1: Fig. S8A). In early stationary phase, substantial amount of GFP-Atg8 entered the vacuolar lumen, masking the signal of potential vacuolar membrane association (Additional file 1: Fig. S8B). In addition, we found that overexpression of Hfl1 in *atg8Δ* cells did not reverse their accumulation of Pho8-positive lumenal structures (Fig. 7c). Conversely, when we artificially tethered Atg8 to the vacuolar membrane in *hfl1Δ* cells by blocking its delipidation (*atg4Δ ATG8ΔR*) (Fig. 7b), the internalization of GFP-Pho8 was largely restored (Fig. 7d). Fusion of Atg8 to a vacuolar membrane protein (Yml018c), however, did not bypass the need of Hfl1 (Additional file 1: Fig. S8C), implying that the function of Atg8 may require a particular structural conformation. Taken together, these data indicate a key function of Hlf1 in EVT is the membrane recruitment of Atg8.

## Discussion

In this study, we reported a potentially general vacuolar membrane protein turnover process, EVT, which occurs when yeast cells approache a nutrient-depleted stationary phase. EVT substrates are vacuolar integral membrane proteins. Upon internalization, these membrane proteins are degraded within the vacuole. EVT does not depend on Atg protein-mediated macroautophagy. Proteins involved in several other autophagic pathways (PMN, VID, etc.) are also dispensable. Instead, EVT employs all the five ESCRT complexes. Compared with other ESCRT mediated pathways, EVT is distinct in its utilization of Atg8 and Hfl1. The recruitment of Atg8 to the vacuolar membrane depends on its interaction with Hfl1. In the presence of Hfl1-Atg8 interaction, the lipidation of Atg8 is not essential. However, forced lipidation of Atg8 bypasses the requirement of Hfl1. These data suggest that EVT is a new variant of autophagy with unique molecular mechanisms.

The vacuolar membrane proteins we examined are not known to function together in a particular pathway. We therefore suspect that the driving signal of EVT is not directed towards specific cargos, but instead exerts a general effect to the vacuolar membrane or certain domains of it. One candidate regulator of EVT is the TORC1 complex. Inhibition of TORC1 has been shown to induce degradation of vacuolar proteins, in part via regulation of Vps27 [21, 51, 52]. The journey towards stationary phase represents one of gradual nutrient depletion, which leads to progressive inhibition of TOCR1 activity [21]. When we were experimenting with conditions causing vacuolar membrane protein degradation, we noticed that switching cells back to fresh medium inhibited GFP-Pho8 translocation. Conversely, using a medium containing moderately reduced levels of major nutrients (1/64 of normal glucose and amino acids) was able to accelerate Pho8 turnover, which still depended on Atg8 and ESCRTs (data not shown). These observations are consistent with nutrient exhaustion being the trigger of EVT and support a role for TORC1 in signal transduction.

Our data indicate that the vacuole is the site where its membrane proteins are internalized (Fig. 5). This implies that EVT occurs as a type of microautophagy. If this is the case, it will be consistent with known function of ESCRTs in driving membrane invaginations. To understand the membrane dynamics in EVT, we tried to characterize the journey of substrate translocation by time-lapse imaging. Entry into stationary phase is a gradual transition that takes many hours. During this period, we saw frequent deformation of vacuoles into cup-shaped structures (data not shown). However, these observations did not provide conclusive evidence that the cups indeed resolved into lumenal vesicles, as the deformations were often reversed. Similar reversals of the membrane deformations have also been documented by other scientists [53, 54]. Further complicating the issue is that the translocation of membrane proteins was retarded when cells were placed on glass-bottom dishes. Thus at present we do not have direct morphological evidence that membrane invagination leads to EVT substrate internalization. To this end, we note that ESCRT depletion increased the level of vacuole fragmentation (Fig. 5a), and that EVT was defective when Vam3 or Vps39 was depleted (data not shown), which point to the vacuole fusion-fission cycle as a potential player [55]. However, EVT was normal in *atg18Δ* cells that displayed substantially reduced level of vacuole fragmentation (Fig. 5a, b), which argues against an essential role of the fusion-fission cycle in EVT. Although the underlying cause remains obscure, the involvement of vesicle fusion factors has also been reported in other microautophagic processes, both in yeast and in mammals [40, 56]. Furthermore, vacuolar lumenal structures were evident in *atg8Δ* cells (Figs. 2 and 3). These vesicle-like structures contained cytosolic materials (Fig. 3c) and depended on ESCRTs for their formation (Fig. 4e), both of which differ from the characters of the intralumenal fragment pathway. Overall, these data favor microautophagy as the most plausible model of EVT substrate translocation.

Our data demonstrate that Atg8 acts downstream of Hfl1 to regulate the formation of intralumenal membrane structures. The main role of Hfl1 in EVT appears to be the recruitment of Atg8 independent of its lipidation (Figs. 6 and 7). In *Arabidopsis*, Hfl1 homologs (LAZ1 and LAZH1) function in the turnover of BAK1, a co-receptor in brassinosteroid signaling [57]. Intriguingly, *laz1 lazh1* cells accumulate BAK1 on vacuoles, which adopt a multi-vesicular morphology resembling that in *hfl1Δ* or *atg8Δ* yeast. These data imply that a EVT-like process may exist in plants, although whether it involves any of the many plant Atg8 homologs remains to be determined. Lipidation-independent roles of Atg8 in the regulation of vacuoles have also been reported by several groups [58, 59], although the significance of Hfl1-Atg8 interaction has only been documented in two yeast systems, *Schizosaccharomyces pombe* and *Saccharomyces cerevisiae* [50]. Interestingly, data from Liu et al. and us both demonstrated that direct fusion of Atg8 to an unrelated vacuolar membrane protein was insufficient to circumvent Hfl1-Atg8 interaction. In *S. pombe*, vacuole morphology in *Sphfl1Δ* cells was restored only when SpAtg8 was fused to a point mutant of SpHfl1. In our case, only Atg8-PE was effective in *hfl1Δ* cells, but not Atg8-Yml018c fusion (Fig. 7a, Additional file 1: Fig. S8C). The precise impact of PE conjugation to Atg8 is not entirely understood. Nevertheless, it is common knowledge in the macroautophagy field that lipidated and non-lipidated Atg8s display differential sensitivities towards antibodies in immunoblot analysis, implying the presence of conformational variations. Thus we suspect that both Hfl1 interaction and PE lipidation may favor a particular Atg8 confirmation. However, what lies downstream of Atg8 likely differs between EVT and macroautophagy. In macroautophagy, the lack of Atg8 constrains membrane expansion [60, 61]. Yet in EVT, the lack of Atg8 leads to the appearance of larger intravacuolar vesicle-like structures (Figs. 2 and 3). In macroautophagy, one main role of Atg8 is the recruitment of cargos or cargo receptors. In EVT, the substrates proteins are already at the membrane and are able to reach lumenal compartments in *atg8Δ* cells, indicating that cargo sorting is unlikely to be the function of Atg8. Lastly, both Hfl1 and Atg8 are important for the organization of vacuolar membrane into lipid microdomains [50], which are involved in microlipophagy. However, we found that when cells approach stationary phase, EVT occurred earlier than microlipophagy, and that EVT was normal in *ncr1Δ npc2Δ* cells in which microdomain formation and microlipophagy were comprised (Additional file 1: Fig. S5), suggesting that a potentially new mechanism is involved.

Historically, the ESCRT machinery and ATG machinery were discovered and investigated separately in the vacuolar protein sorting/endosomal multivesicular body pathway and the macroautophagy pathway. In recent years, the line between these two sets of protein machinery is getting blurred. Reports from several research groups have shown that the ESCRT machinery is critical in the sealing of autophagosomal membrane [62–64]. In yeast, a component of the initiating scaffold, Atg17, recruits ESCRT III subunit Snf7 to the site of autophagosome formation [64]. The expected timing of the sealing step also places the action of ESCRT at a later stage in macroautophagy. In the present study, we identified a key Atg protein acting downstream of ESCRTs in a microautophagy-like process, expanding the possibilities for the interplay between ESCRTs and ATGs. Currently, other than their epistatic relationship, we do not know how ESCRTs and the Hfl1-Atg8 pair is connected. It is also not clear how a protein as small as Atg8 can participate in these and other seemingly distinct processes. Future studies will be needed to answer these important questions and to better understand the principles underlying complex membrane dynamics.

## Conclusion

Entry into stationary phase triggers the internalization and degradation of vacuolar intergral membrane proteins. Their internalization depends on the ESCRT complexes and the membrane recruitment of Atg8 by Hfl1, but not most other Atg proteins. Their degradation is mediated by vacuolar hydrolases.

## Methods

Unless otherwise noted, all experiments in this study were repeated at least 3 times, with representative results shown in figures.

### Culturing of yeast cells

Yeast culturing was done at 30 °C.

For most experiments examining the EVT process, cells were inoculated into SMD+CA(0.67% yeast nitrogen base without amino acids, 0.5% casamino acid, 2% glucose, adenine 30 mg/L, histidine 20 mg/L, leucine 50 mg/L, tryptophan 50 mg/L, lysine 30 mg/L, uracil 20 mg/L, methionine 30 mg/L) and cultured overnight. On the morning of the second day, cells were diluted to OD_600_ = 0.2 and cultured for another 8 h or 32 h before collection (or 56 h, for lipophagy).

When noted in the text, 6 h after cell culture was diluted to OD_600_ = 0.2, manipulations to deplete AID tagged proteins, to shut off tetO controlled expression, or to induce P_CUP1_ controlled expression were initiated by the addition of 0.5 mM IAA, 1 μg/ml doxycycline, or 30 μM CuSO_4_, respectively. Note that the use of copper to enhance expression was only employed in Fig. 5d. Cells were cultured for another 8 h before collection.

For experiments involving P_GAL_ controlled GFP-Cps1, cells were initially cultured in SMR+CA(0.67% yeast nitrogen base without amino acids, 0.5% casamino acid, 2% raffinose, adenine 30 mg/L, histidine 20 mg/L, leucine 50 mg/L, tryptophan 50 mg/L, lysine 30 mg/L, uracil 20 mg/L, methionine 30 mg/L). When indicated, 500 μM IAA was added to the medium when culture reached OD_600_ = 2.0. After another 2.5 h, 1% galactose was added to induce gene expression for 1 h (Cps1).

For experiments examining PMN, cells were inoculated in SMD+CA. On the morning of the second day, cells were diluted to OD_600_ = 0.2 and cultured for another 24 h. Cells were then shifted to SD-N medium (2% glucose, 0.17% yeast nitrogen base without amino acids and ammonium sulfate) for 8 h.

For experiments examining VID, cells were grown in YPKG medium (1% yeast extract, 2% peptone, 1% potassium acetate, and 0.5% glucose) for 3 days. Cells were then transferred to YPD medium (1% yeast extract, 2% peptone, and 2% glucose) medium for another 3 h.

### Fluorescent microscopy

Glass-bottom dishes were coated with 1 mg/ml Concanavalin A to immobilize yeast cells immersed under SMD-CA or water. Ambient temperature was approximately 25–28 °C. Images were acquired on an Olympus IX83 microscope equipped with a Hamamatsu ORCA-Flash 4.0 LT camera, controlled by Micro-Manager 1.4 software. The objective lens used was UPLSAPO100XO (100X/1.4). For Z-stacks, 15 sections were collected with a stepping size of 0.5 μm. For FM4-64 staining, cells were incubated with 2 μM FM4-64 for 5 min and washed and cultured for another 0.5 h (log phase) or 3 h (early stationary phase) before imaging.

### Transmission electron microscopy (TEM)

TEM sample preparation was performed as previously described [65] with the following three modifications: (1) SPI-Pon 812 was used as the embedding resin; (2) concentrations of resin during infiltration were 33%, 66%, and then 100% three times; and (3) post-embedding staining was omitted.

### Protein immunoprecipitation

Yeast cells were washed by water and Lysis Buffer (50 mM Tris-Cl, 150 mM NaCl, 10% v/v glycerol, 1 mM EDTA, 1 mM PMSF, 0.2% v/v NP-40, pH 7.4) once each. Cells were then suspended in Lysis Buffer, chilled on ice, and lysed by glass beads on a bead mill. Supernatants were collected after centrifugation at 10,000*g* at 4C for 5 min. Anti-GFP affinity resin (Smart Lifescience) was washed three times in Wash Buffer (50 mM Tris-Cl, 150 mM NaCl, 10% v/v glycerol, 1 mM EDTA, pH 7.4). Affinity resin was then loaded with yeast cell lysates and incubated at 4C with gentle rotation for 2 h. Afterwards, affinity resin was washed three times in Wash Buffer and processed for SDS-PAGE and immunoblotting.

### Protein expression, purification, and differential scanning calorimetry (DSC)

GST-Atg8 was produced in an *E. coli* BL-21 strain. Bacteria were cultured at 37 °C until OD600 reached 0.6. The culture was supplemented with 1 mM IPTG and incubated at 18 °C for 12 h to induce protein expression. Cells were collected by centrifugation, resuspended in PBS buffer, and then lysed using a high-pressure cell lysis machine (Union-Biotech, UH-24). After cell debris was cleared by centrifugation, the lysate was incubated with glutathione resin (Beyotime, P2251) at 4 °C for 2 h. After centrifugation, the resin was collected and washed with proteolysis buffer (50 mM Tris-HCl, 150 mM NaCl, 1 mM EDTA, 1 mM DTT, pH 7.5) for five times. The resin was then incubated in proteolysis buffer in the presence of 3C protease (Beyotime, P2303) for 16 h at 4 °C to release Atg8. This was followed by a second purification step using a molecular sieve column (Uniondex 75 pg 16/60, Union-Biotech, US75) on a FPLC (Union-Biotech, UEV 25D). The sample containing Atg8 was eluted in proteolysis buffer without DTT. The sample was finally concentrated using a centrifugal filter (Amicon Ultra-15, Millipore, UFC900308). Protein melting temperature was determined on a differential scanning calorimeter (MicroCal VP-Capillary DSC, GE Healthcare) following the manufacturer’s instructions. The concentrations of Atg8 proteins were adjusted to 0.2–0.3 mg/ml for DSC.

### Other methods

Assays for macroautophagy were performed as previously described [66].

### Strains and plasmids

Plasmids used in this study are listed in Table S1, S2, & S3 [12, 48, 67–73]. For strain construction, gene knockout was performed using the conventional PCR-based method. The primers used are listed in Table S4. Strains used in this study are listed in Table S5 [74].

## Supplementary Information

**Additional file 1: Figure S1.** EVT is a general process for integral vacuolar membrane proteins. **Figure S2.** Testing the potential role of Atg proteins in EVT. **Figure S3.** EVT is distinct from PMN and VID. **Figure S4.** EVT does not require the VTC complex and the EGO complex. **Figure S5.** EVT is distinct from NPC-dependent lipophagy. **Figure S6.** The role of ESCRT proteins in EVT. **Figure S7.** Hfl1 interacts and functions together with Atg8. **Figure S8.** Relationship between Hfl1 and Atg8.

**Additional file 2: Table S1.** Plasmids Set I. **Table S2.** Plasmids Set II. **Table S3.** Plasmids Set III. **Table S4.** Primers for gene knockout and tagging. **Table S5.** Strains used in this study.

**Additional file 3.** Raw Data. Raw data in excel file for all column graphs in figures and additional file figures.

**Additional file 4.** Uncropped Blots. Uncropped images for all immunoblots in figures and additional file figures.

## Data Availability

All supporting data in this study are provided in the main article or the associated additional files.

## Abbreviations

AID: Auxin-inducible degron
Atg: Autophagy-related
ESCRT: Endosomal sorting complex required for transport
GFP: Green fluorescent protein
MVB: Multi-vesicular body
PE: Phosphatidylethanolamine
PMN: Piecemeal microautophagy of the nucleus
VID: Vacuole import and degradation
