# Supplementary material for: Membrane recruitment of Atg8 by Hfl1 facilitates turnover of vacuolar membrane proteins in yeast cells approaching stationary phase
Source: BMC Biol. 2021 Jun 4;19:117. doi: 10.1186/s12915-021-01048-7 (PMC8176713; doi:10.1186/s12915-021-01048-7)
Supplement: Supplementary file 1 — Additional file 1: Figure S1. EVT is a general process for integral vacuolar membrane proteins. Figure S2. Testing the potential role of Atg proteins in EVT. Figure S3. EVT is distinct from PMN and VID. Figure S4. EVT does not require the VTC complex and the EGO complex. Figure S5. EVT is distinct from NPC-dependent lipophagy. Figure S6. The role of ESCRT proteins in EVT. Figure S7. Hfl1 interacts and functions together with Atg8. Figure S8. Relationship between Hfl1 and Atg8. [file 12915_2021_1048_MOESM1_ESM.pdf]

Figure S1

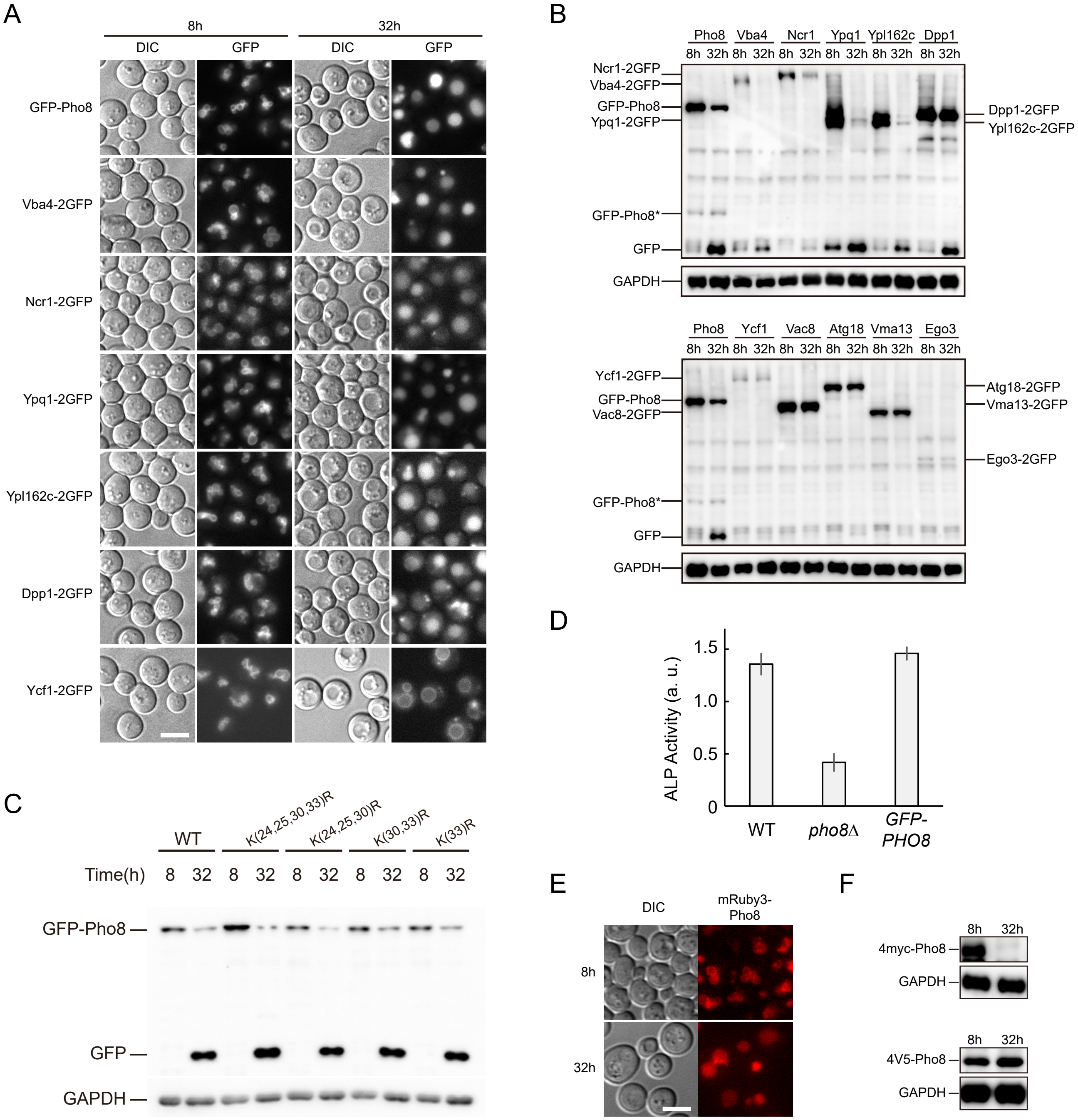

A
8h
32h
DIC
GFP
DIC
GFP
GFP-Pho8
Vba4-2GFP
Ncr1-2GFP
Ypq1-2GFP
Ypl162c-2GFP
Dpp1-2GFP
Ycf1-2GFP
B
Pho8
Vba4
Ncr1
Ypq1
Ypl162c
Dpp1
8h 32h 8h 32h 8h 32h 8h 32h 8h 32h 8h 32h
Ncr1-2GFP
Vba4-2GFP
GFP-Pho8
Ypq1-2GFP
GFP-Pho8*
GFP
GAPDH
Dpp1-2GFP
Ypl162c-2GFP
Pho8
Ycf1
Vac8
Atg18
Vma13
Ego3
8h 32h 8h 32h 8h 32h 8h 32h 8h 32h 8h 32h
Ycf1-2GFP
GFP-Pho8
Vac8-2GFP
GFP-Pho8*
GFP
GAPDH
Atg18-2GFP
Vma13-2GFP
Ego3-2GFP
C
WT
K(24,25,30,33)R
K(24,25,30)R
K(30,33)R
K(33)R
Time(h) 8 32 8 32 8 32 8 32 8 32
GFP-Pho8
GFP
GAPDH
D
ALP Activity (a. u.)
1.5
1.0
0.5
0
WT
pho8Δ
GFP-PHO8
E
DIC
mRuby3-Pho8
8h
32h
F
8h 32h
4myc-Pho8
GAPDH
8h 32h
4V5-Pho8
GAPDH

Figure S1. EVT is a general process for integral vacuolar membrane proteins.

(A) Most integral vacuolar proteins translocate to the vacuolar lumen during early stationary phase. Cells treated and image presented as in Fig. 1A.

(B) Most integral vacuolar proteins, but not peripheral vacuolar proteins, are subject to degradation in early stationary phase. Vac8, Atg18, Vma13, and Ego3 are peripheral membrane proteins. Cells treated and image presented as in Fig. 1B.

(C) Lysines at the cytosolic domain of Pho8 is dispensable for its turnover. The *PHO8* locus was replaced with *pho8* mutants carrying lysine-arginine substitutions as indicated. Cells treated and image presented as in Fig. 1B.

(D) GFP-Pho8 is functional as an alkaline phosphatase (ALP). The ALP activity in WT, *pho8Δ*, and *pho8Δ* cells expressing GFP-Pho8 was quantified. A. u., arbitrary units. Error bar, standard deviation, n = 5.

(E) Translocation of mRuby3-Pho8 in early stationary phase. Cells treated and image presented as in Fig. 1A.

(F) Turnover of 4myc-Pho8, but not 4V5-Pho8, in early stationary phase. Cells treated and image presented as in Fig. 1B.

Figure S2

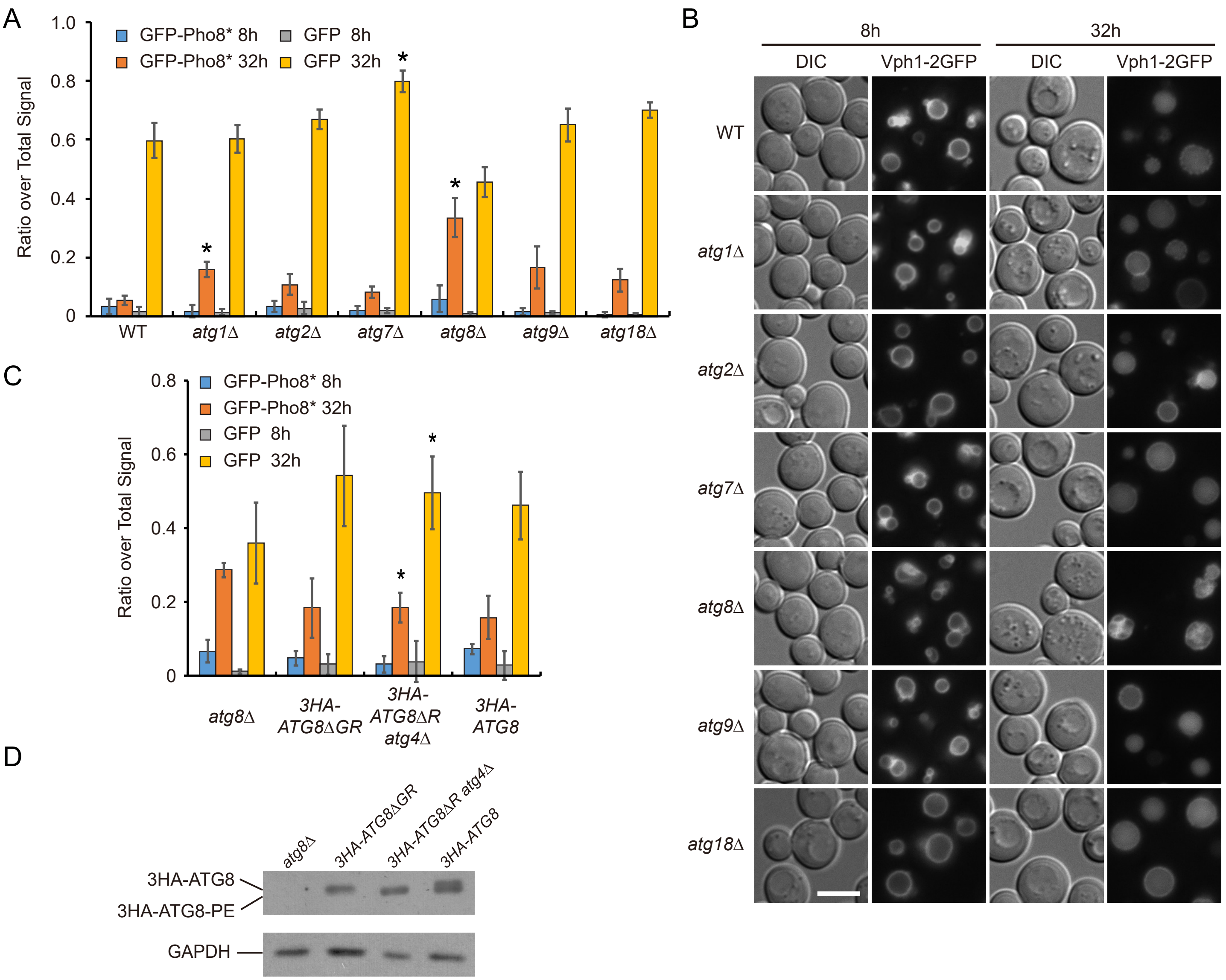

A
Ratio over Total Signal
GFP-Pho8* 8h
GFP-Pho8* 32h
GFP 8h
GFP 32h
WT
atg1Δ
atg2Δ
atg7Δ
atg8Δ
atg9Δ
atg18Δ
B
8h
32h
DIC
Vph1-2GFP
C
3HA-ATG8ΔGR
3HA-ATG8ΔR atg4Δ
3HA-ATG8
D
3HA-ATG8
3HA-ATG8-PE
GAPDH

Figure S2. Testing the potential role of Atg proteins in EVT.

(A) Quantification of GFP-Pho8 turnover in *atg* mutants. The experiment was repeated three times. Representative immune-blot is presented in Fig. 3B. The ratios of GFP-Pho8* and free GFP over the sum of GFP-Pho8, GFP-Pho8* and free GFP are shown. Error bar, standard deviation, n = 3. *, t-test in comparison with WT shows significance value < 0.05.

(B) The translocation of Vph1-2GFP into vacuolar lumen involves a conjugation-independent function of Atg8. Cells treated and image presented as in Fig. 1A.

(C) Quantification of GFP-Pho8 turnover in *atg8* mutants. Representative immune-blot is presented in Fig. 3D. Quantification data presented as in A. *, t-test in comparison with *atg8Δ* shows significance value < 0.05.

(D) Immuno-blots showing the conjugation status of different Atg8 variants. Atg8ΔGR is completely defective in PE conjugation. Atg8ΔR is stuck at the conjugated state in *atg4Δ* cells.

Figure S3

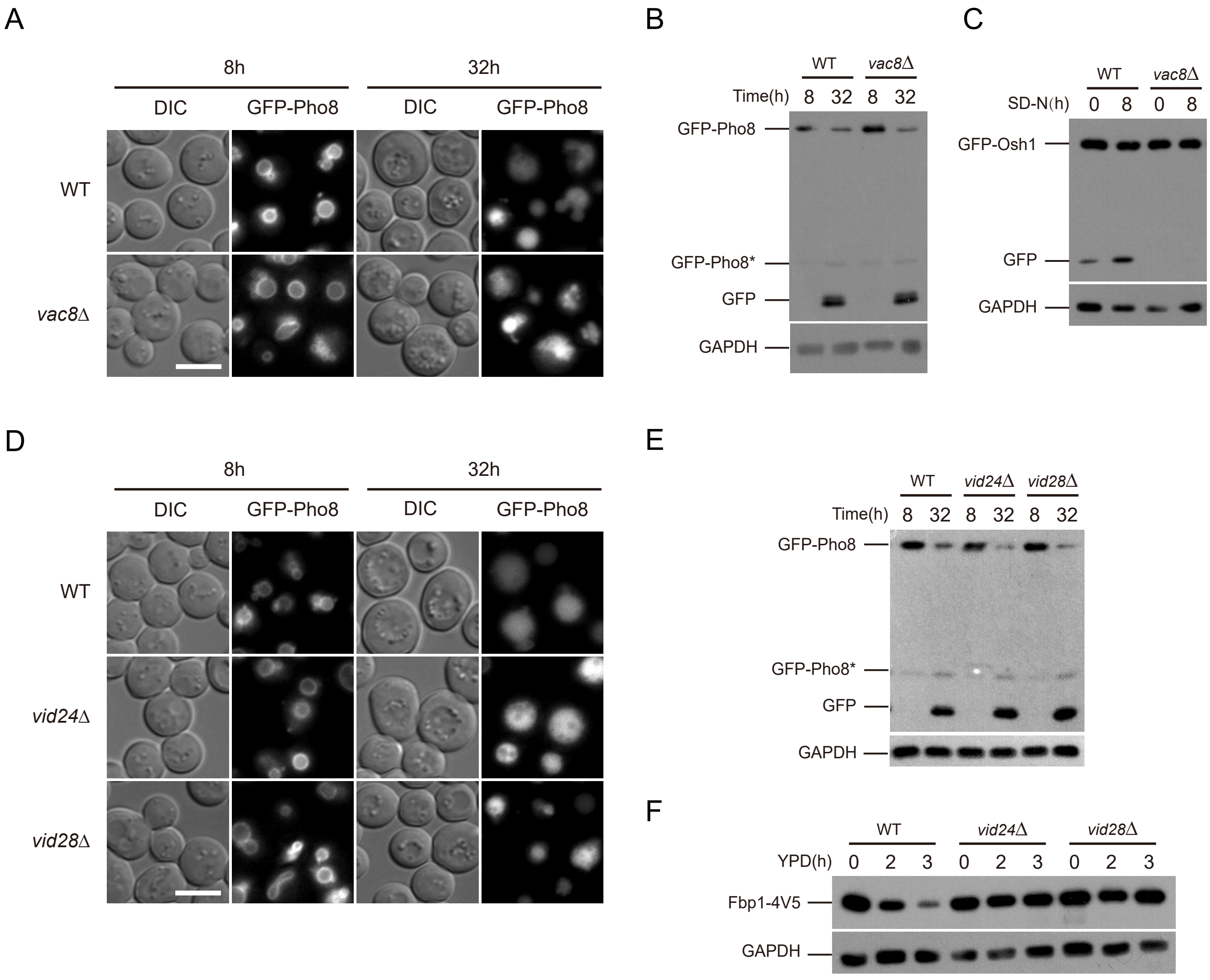
A
8h
32h
DIC
GFP-Pho8
DIC
GFP-Pho8
WT
vac8Δ
B
WT
vac8Δ
Time(h) 8 32 8 32
GFP-Pho8
GFP-Pho8*
GFP
GAPDH
C
WT
vac8Δ
SD-N(h) 0 8 0 8
GFP-Osh1
GFP
GAPDH
D
8h
32h
DIC
GFP-Pho8
DIC
GFP-Pho8
WT
vid24Δ
vid28Δ
E
WT
vid24Δ
vid28Δ
Time(h) 8 32 8 32 8 32
GFP-Pho8
GFP-Pho8*
GFP
GAPDH
F
WT
vid24Δ
vid28Δ
YPD(h) 0 2 3 0 2 3 0 2 3
Fbp1-4V5
GAPDH

Figure S3. EVT is distinct from PMN and VID.

(A, B) The degradation of GFP-Pho8 is independent of *VAC8*. Cells treated and data presented as in Fig. 1A and 1B, respectively.

(C) Control experiment showing that *vac8Δ* cells are defective in PMN. Log phase yeast cultures were diluted to OD_{600}=0.2 and grown for another 24 h. Cells were then shifted to nitrogen-starvation medium (SD-N) for 8 h. The turnover of GFP-Osh1 (a PMN substrate) was monitored by immuno-blot.

(D, E) The degradation of GFP-Pho8 is independent of *VID24* and *VID28*. Cells treated and data presented as in Fig. 1A and 1B, respectively.

(F) Control experiment showing that *vid* mutants are defective in VID. Cells were first grown in a low glucose medium (YPKG) for 3 days. Cells were then transferred to a medium containing normal amount of glucose (YPD) for another 3 h. The turnover of Fbp1 (a VID substrate) was monitored by immuno-blot.

Figure S4

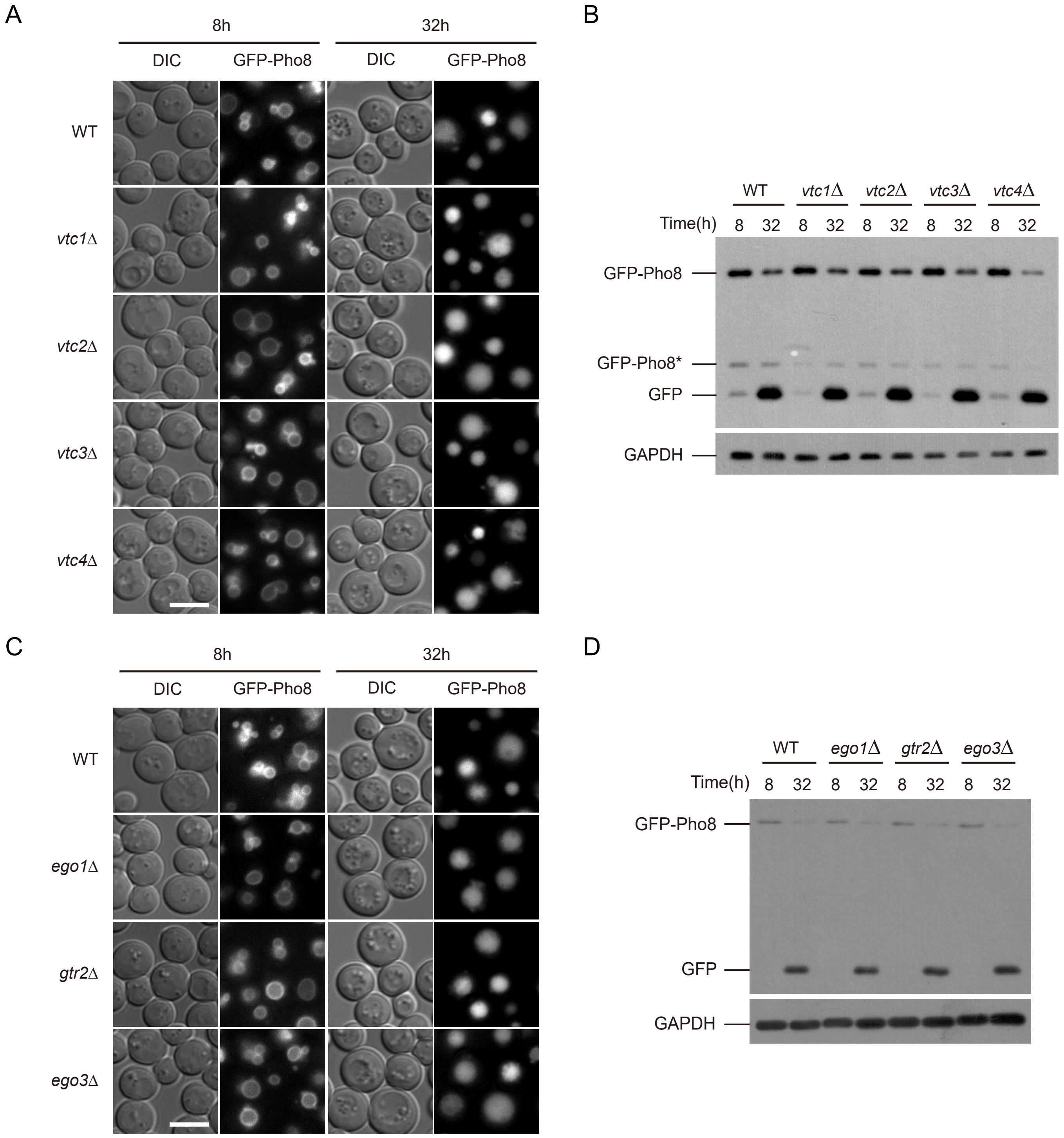
A
8h
32h
DIC
GFP-Pho8
DIC
GFP-Pho8
WT
vtc1Δ
vtc2Δ
vtc3Δ
vtc4Δ
B
WT
vtc1Δ
vtc2Δ
vtc3Δ
vtc4Δ
Time(h)
8 32 8 32 8 32 8 32 8 32
GFP-Pho8
GFP-Pho8*
GFP
GAPDH
C
8h
32h
DIC
GFP-Pho8
DIC
GFP-Pho8
WT
ego1Δ
gtr2Δ
ego3Δ
D
WT
ego1Δ
gtr2Δ
ego3Δ
Time(h)
8 32 8 32 8 32 8 32
GFP-Pho8
GFP
GAPDH

Figure S4. EVT does not require the VTC complex and the EGO complex.

(A, B) The degradation of GFP-Pho8 in VTC complex mutants is normal. Cells treated and data presented as in Fig. 1A and 1B, respectively.

(C, D) The degradation of Pho8 in EGO complex mutants is normal. Cells treated and data presented as in Fig. 1A and 1B, respectively.

Figure S5

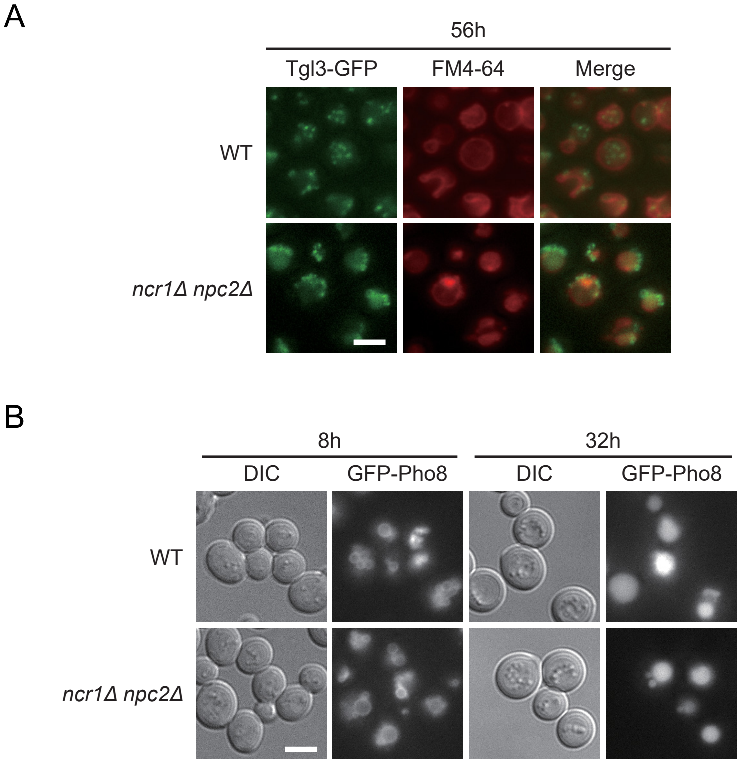

A
56h
Tgl3-GFP
FM4-64
Merge
WT
ncr1Δ npc2Δ
B
8h
32h
DIC
GFP-Pho8
DIC
GFP-Pho8
WT
ncr1Δ npc2Δ

Figure S5. EVT is distinct from NPC-dependent lipophagy.

(A) Lipophagy occurs later than EVT and depends on *NCR1* and *NPC2*. Cells of the indicated genotypes were treated as in Fig. 1E and observed 56 h after dilution to OD_{600}=0.2.

(B) The translocation of GFP-Pho8 to vacuolar lumen is normal in *ncr1Δ npc2Δ* cells. Cells of the indicated genotypes were treated as in Fig. 1A and observed at the indicated time points.

Figure S6

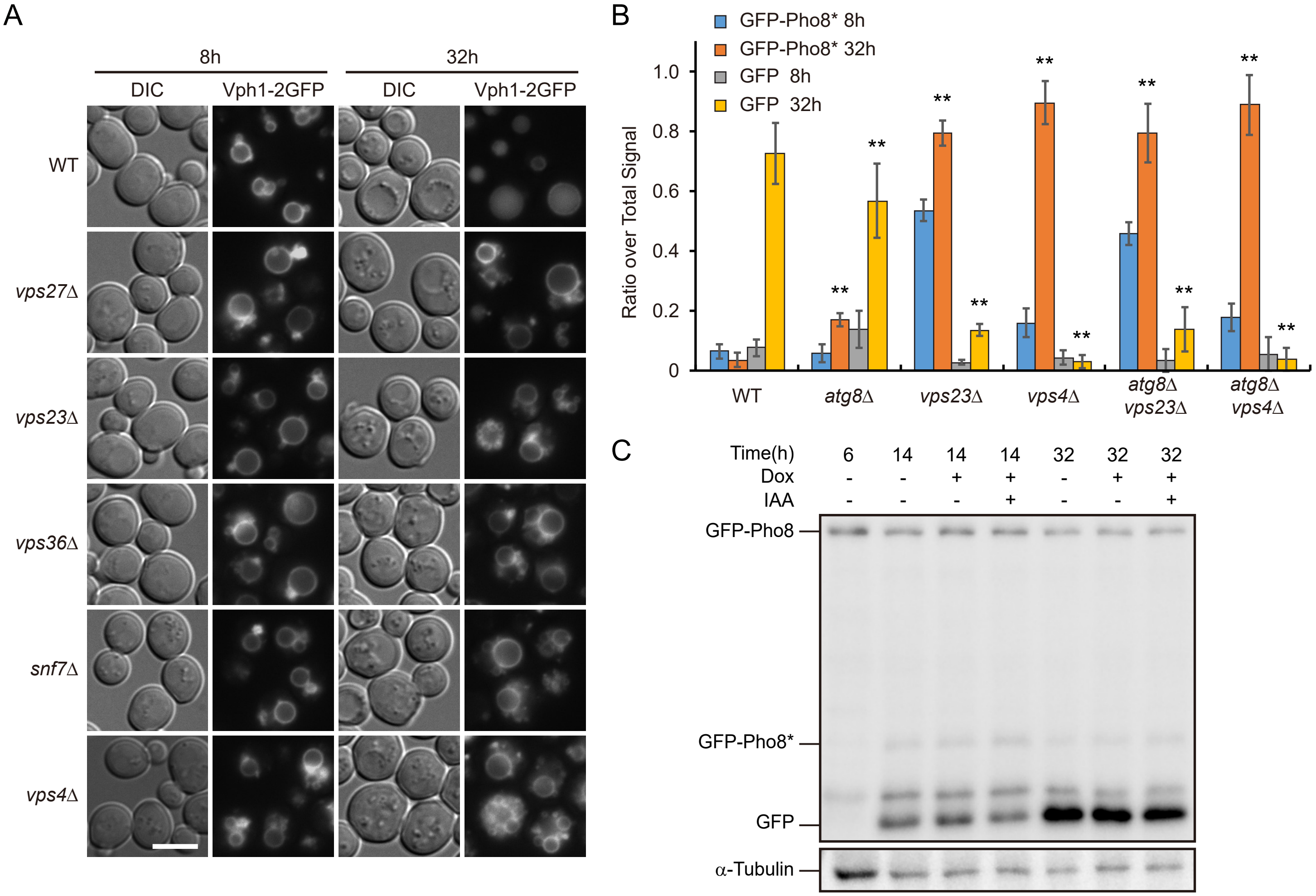
A
8h
32h
DIC
Vph1-2GFP
DIC
Vph1-2GFP
WT
vps27Δ
vps23Δ
vps36Δ
snf7Δ
vps4Δ
B
GFP-Pho8* 8h
GFP-Pho8* 32h
GFP 8h
GFP 32h
Ratio over Total Signal
1.0
0.8
0.6
0.4
0.2
0
**
WT
atg8Δ
vps23Δ
vps4Δ
atg8Δ vps23Δ
atg8Δ vps4Δ
C
Time(h) 6 14 14 14 32 32 32
Dox - - + + - + +
IAA - - - + - - +
GFP-Pho8
GFP-Pho8*
GFP
α-Tubulin

Figure S6. The role of ESCRT proteins in EVT.

(A) The degradation of Vph1-2GFP is defective in ESCRT mutants. Cells treated and data presented as in Fig. 1A.

(B) Quantification of GFP-Pho8 turnover. Representative immune-blot is presented in Fig. 4F. Quantification data presented as in A. n = 4. ***, t-test in comparison with WT shows significance value < 0.01.

(C) Turnover of GFP-Pho8 is detectable at an early time. Cells treated as in Fig. 5A. Samples at three different time points were collected and analyzed by immune-blotting.

Figure S7

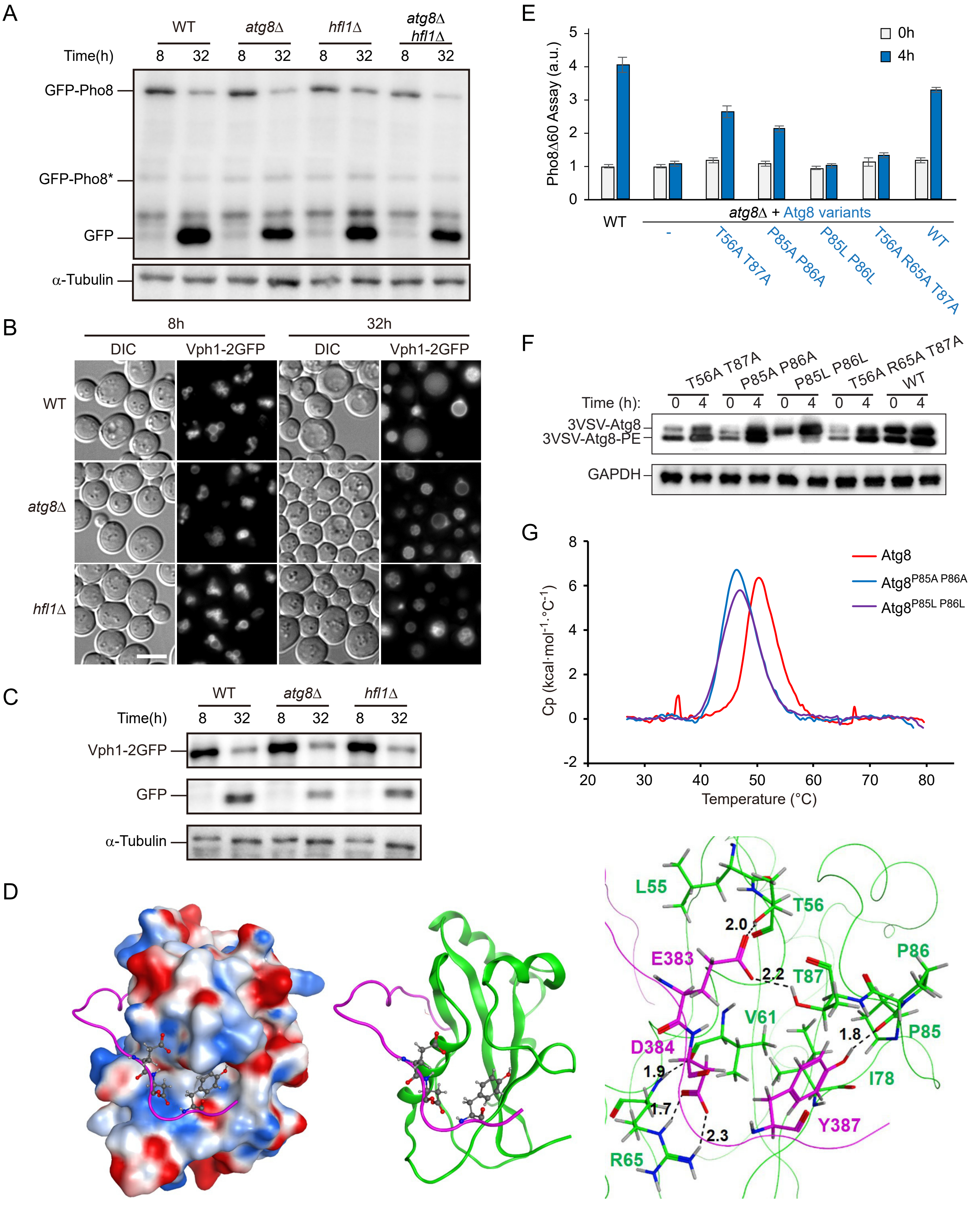

A
WT
atg8Δ
hfl1Δ
atg8Δ hfl1Δ
Time(h)
8 32 8 32 8 32 8 32
GFP-Pho8
GFP-Pho8*
GFP
α-Tubulin
B
8h
32h
DIC
Vph1-2GFP
WT
atg8Δ
hfl1Δ
C
WT
atg8Δ
hfl1Δ
Time(h)
8 32 8 32 8 32
Vph1-2GFP
GFP
α-Tubulin
D
L55
T56
2.0
E383
2.2
T87
P86
V61
D384
1.8
P85
1.9
I78
1.7
Y387
2.3
R65
E
Pho8Δ60 Assay (a.u.)
0h
4h
WT
atg8Δ + Atg8 variants
-
T56A T87A
P85A P86A
P85L P86L
T56A R65A T87A
WT
F
T56A T87A
P85A P86A
P85L P86L
T56A R65A T87A
WT
Time (h):
0 4 0 4 0 4 0 4 0 4
3VSV-Atg8
3VSV-Atg8-PE
GAPDH
G
Cp (kcal·mol-1·°C-1)
Temperature (°C)
Atg8
Atg8P85A P86A
Atg8P85L P86L

Figure S7. Hfl1 interacts and functions together with Atg8.

(A) Processing of GFP-Pho8 into free GFP is reduced in *hfl1Δ* cells and *atg8Δ hfl1Δ* cells, similar to *atg8Δ* cells. Cells treated and image presented as in Fig. 1B.

(B) Vph1-2GFP accumulates on intravacuolar structures in *atg8Δ* cells and *hfl1Δ* cells. Cells treated and image presented as in Fig. 1A.

(C) Processing of Vph1-2GFP into free GFP is reduced in in *atg8Δ* cells and *hfl1Δ* cells. Cells treated and image presented as in Fig. 1B.

(D) Critical interactions between residues of Hfl1 and Atg8 at the Y site. **Right image:** Residues from Hfl1 and Atg8 are colored as magenta and green, respectively. Key hydrogen bonds are indicated using dashed lines, with distances (Angstrom) labeled in back. **Left and middle images:** The whole Atg8 protein is presented in space-fill and ribbon diagrams to illustrate the context of the key interacting amino acids, with the backbone of Hfl1 C-terminus shown in magenta, and E383, D384 and Y387 of Hfl1 highlighted in balls and sticks. The model is based on PDB structure 6AAG, using *Saccharomyces cerevisiae* proteins.

(E-F) Atg8 mutants defective in EVT are also defective in macroautophagy. Cells were grown to mid-log phase, then shifted to nitrogen starvation medium for 4 h. **(E)** Autophagic flux measured in the pho8Δ60 assay. Error bar, standard deviation, n = 3. **(F)** Conjugation status of Atg8 analyzed by immuno-blotting.

(G) P85A P86A and P85L P86L mutants of Atg8 have reduced thermostability. Wild-type, P85A P86A, and P85L P86L variants of Atg8 were expressed in E. coli and purified. Their thermostabilities were measured by differential scanning calorimetry (DSC). The experiments were repeated twice. One representative result is shown. Cp, heat capacity.

Figure S8

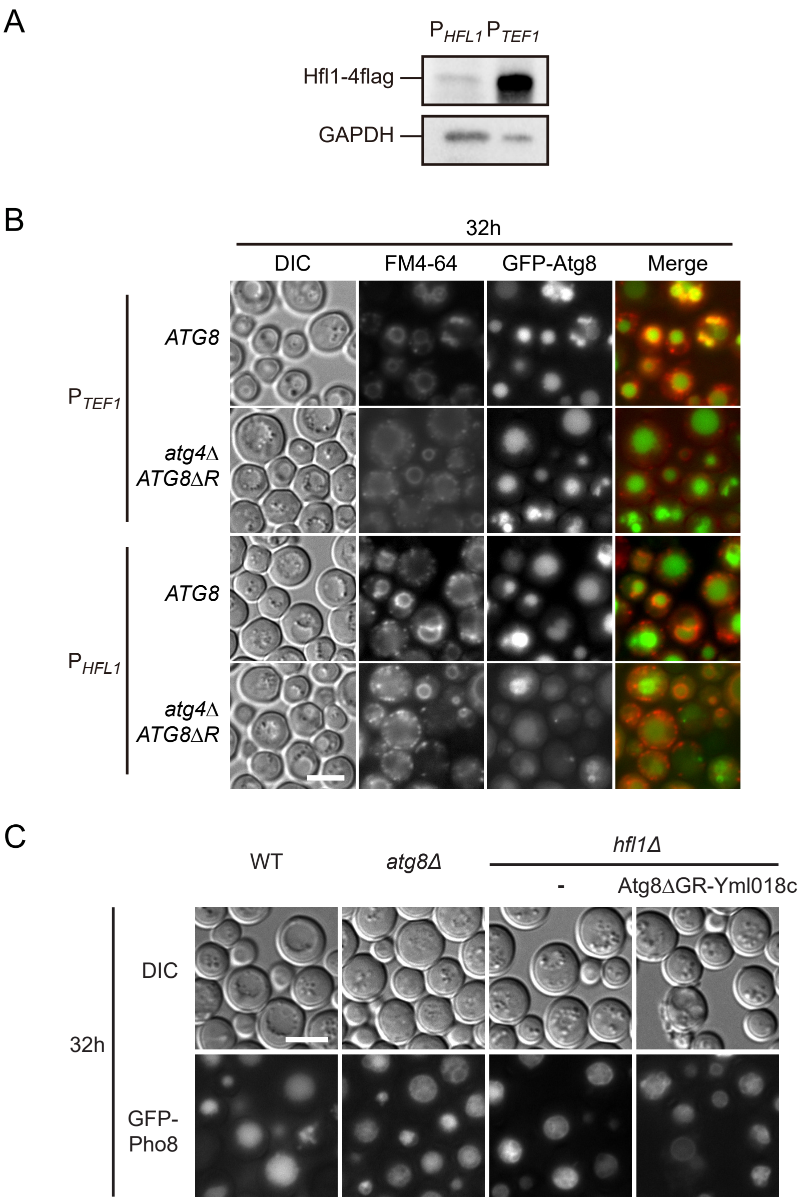

A
P HFL1
P TEF1
Hfl1-4flag
GAPDH
B
32h
DIC
FM4-64
GFP-Atg8
Merge
P TEF1
ATG8
atg4Δ
ATG8ΔR
P HFL1
ATG8
atg4Δ
ATG8ΔR
C
WT
atg8Δ
hfl1Δ
-
Atg8ΔGR-Yml018c
32h
DIC
GFP-
Pho8

Figure S8. Relationship between Hfl1 and Atg8.

(A) Immunoblot showing the level of Hfl1 overexpression under the *TEF1* promoter.

(B) Translocation of GFP-Atg8 into vacuolar lumen in early stationary phase. The strong lumenal GFP signal made it difficult to assess the association of Atg8 with the vacuolar membrane. Cells treated and image presented as in Fig. 7B.

(C) Fusion of Atg8 to a vacuolar membrane protein does not bypass *hfl1Δ*. Atg8 was fused to a vacuolar membrane protein, Yml018c. The omission of the last two amino acids (G116 and R117) in Atg8 ensures that the fusion is not cleavable by Atg4. Translocation of GFP-Pho8 was assessed as in Fig.1A.
