# Supplementary material for: Membrane recruitment of Atg8 by Hfl1 facilitates turnover of vacuolar membrane proteins in yeast cells approaching stationary phase
Source: BMC Biol. 2021 Jun 4;19:117. doi: 10.1186/s12915-021-01048-7 (PMC8176713; doi:10.1186/s12915-021-01048-7)
Supplement: Supplementary file 2 — Additional file 2: Table S1. Plasmids Set I. Table S2. Plasmids Set II. Table S3. Plasmids Set III. Table S4. Primers for gene knockout and tagging. Table S5. Strains used in this study. [file 12915_2021_1048_MOESM2_ESM.pdf]

## Table S1. Plasmid Set I*.

*Fragments from parental plasmids were obtained by restriction enzyme digestion.

| Plasmid | Parental Plasmid | Inserted Fragments | Restriction Sites | Primers | Templates /Sources |
|---|---|---|---|---|---|
| $P_{CUP1}$-GFP-Pho8-406 | $P_{CUP1}$-GFP-Atg8-406 [12] | Pho8 | BsrGI, KpnI | ATGGATGAATTGTACAAAGGAGCCGGAACAAGTGACATGATGACTCACACATTACC, ACTATAGGGCGAATTGGGTACCGCGCTTCACTTAACGACGAT | Genomic DNA |
| $P_{CUP1}$-GFP-Osh1-406 | $P_{CUP1}$-GFP-Atg8-406 | GFP | SpeI, KpnI | ATATAGAAGTCATCGACTAGTAAC, GATAGATCAGGTTGTTCCATGTCACTTGTTCCGGCTCCTT | $P_{CUP1}$-GFP-Atg8-406 |
| | | Osh1 | | ATGGAACAACCTGATCTATCG, CTATAGGGCGAATTGGGTACCATCGGCTTTGAATCCATGGG | Genomic DNA |
| $P_{CUP1}$-GFP-Cps1-406 | $P_{CUP1}$-GFP-Atg8-406 | Cps1 | BsrGI, KpnI | ATGGATGAATTGTACAAAGGAGCCGGAACAAGTGACATGATCGCCTTACCAGTAGA, GAATTGGGTACCCTTTACGCAAGTGGTTTTGA | Genomic DNA |
| Vph1-2×GFP-URA(K.l.) | KT209-2×GFP-URA3(K.l.) [67] | Vph1(ORF+ promoter) | HindIII | AACGCGGCCGCCAGCTGAAGCTTGCTAGCTTTTACCTTTCTCT, GTCGACCTGCAGCGTACGAAGCGCTTGAAGCGGAAGAGCTTG | Genomic DNA |
| | | Vph1(terminator) | SpeI | TCGATGATATCAGATCCACTAGTGAGGACTTTTAAAAAAAGCGA, TCGATGATATCAGATCCACTAGTTAAATTTTGAGGACTTTTAAAAA | Genomic DNA |
| Ypq2-2×GFP-URA(K.l.) | KT209-2×GFP-URA3(K.l.) | Ypq2(ORF+ promoter) | HindIII | AACGCGGCCGCCAGCTGAAGCTTGGTACCAGTGTTATCACTAAG, GTCGACCTGCAGCGTACGAAGCTGTTTGCGTTTCACCAGACA | Genomic DNA |
| | | Ypq2(terminator) | SpeI | TCGATGATATCAGATCCACTAGTTAGTTTTCCCTACGGAGTACT, CCGCGGCCGCATAGGCCACTAGTCTCGAGCAGTTGGGTCCAC | Genomic DNA |
| BS-TRP1-TetO7-GFP-Pho8 | BS-TRP1 [67] | TetO7 | HindIII | GAGGTCGACGGTATCGATAAGCTTGAATTCTTATTACGATCCTCG, GAATAATTCTTCACCTTTAGACATCGGCCGCTATCGATGTTAA | CM189 [68] |
| | | GFP-Pho8 | | ATGTCTAAAGGTGAAGAATTATTC, CTGCAGGAATTCGATATCAAGCTTCACTTAACGACGATGCCGAT | $P_{CUP1}$-GFP-Pho8-406 |

| | | | | | |
|---|---|---|---|---|---|
| BS-URA3-TetR'-Ssn6 | BS-URA3 [67] | TetR'-Ssn6 | SpeI | GCAGCCCGGGGGATCCACTAGTCGCCATGTTGACATTGATTA, TTGCGGCCGCTCTAGAACTAGTAAGGATACGAAAATGCACCT | CM244 [69] |
| ClhN-URA3(K.l.) | UG72 | T1 | PstI | ACGCTGCAGGTCGTGGGATTCCTGGGTTAATTAACATCGCTAGCCCTAGGTGAGGCGCGCCACTTCTAA, GACCTGCAGATATTACCCTGTTATCCCTAGCGG | Fa6A-3HA-TRP [70] |
| ClhN-MET15 | ClhN-URA3(K.l.) | MET15 | XbaI, SacI | TTAGGTCTAGATGCGAATTAACACTGTTCTT, CTCGAGAGCTCCATTTAAATCCAATTCTTCC | Genomic DNA |
| $P_{ATG8}$-MET15 | ClhN-MET15 | $P_{ATG8}$ | NheI, AvrII | GGGTTAATTAACATCGCTAGCCCTTAAAAACTCCATTGAAGAC, GAAGTGGCGCGCCTCACCTAGGGTCTCTAGTAATTATTTTAT | Genomic DNA |
| ClhN-HIS5(S.p.) | UG27 | T1 | PstI | ACGCTGCAGGTCGTGGGATTCCTGGGTTAATTAACATCGCTAGCCCTAGGTGAGGCGCGCCACTTCTAA, GACCTGCAGATATTACCCTGTTATCCCTAGCGG | Fa6A-3HA-TRP |
| ClhN-2flag-HIS5(S.p.) | ClhN-flag-HIS5(S.p.) | flag | NheI, AvrII, PvuI | (N/A) | ClhN-flag-HIS5(S.p.) |
| ClhN-4flag-HIS5(S.p.) | ClhN-2flag-HIS5(S.p.) | flag | NheI, AvrII, PvuI | (N/A) | ClhN-flag-HIS5(S.p.) |
| IAA17-4flag-HIS5(S.p.) | ClhN-4flag-HIS5(S.p.) | IAA17 | PacI, NheI | TCGTGGGATTCCTGGGTTAATTAACATGATGGGCAGTGTCGAGCT, CGATGTTAATTAACCCGCTAGCAGCTCTGCTCTTGCACTTCT | MK43-BYP6740 [48] |
| HIS5(S.p.)-$P_{VAC8}$-IAA17-4flag | ClhN-HIS5(S.p.) | $P_{VAC8}$, IAA17-4flag | EcoRV, SacII | ATACGAAGTTATTAGGTGATATAGTGTATCGTCGCTGGAA, TCAGCTCGACACTGCCCATCATACCCATAGTTGCAAATGTAT | Genomic DNA |
| | | | | ACTATGGGTATGATGGGCAGTGTCGAGCTG, AGGCCACTAGTGGATCTGATCCTAGGTTTATCATCATCAT | IAA17-4flag-HIS5(S.p.) |
| $P_{ADH1}$-TIR1-9myc-MET15 | ClhN-MET15 | $P_{ADH1}$-TIR1-9myc | NheI | TGGGTTAATTAACATCGCTAGCAATTCGGGTGTACAATATGG, GCGCGCCTCACCTAGGGCTAGCCTTTTTAGCTAGTGGATCCG | NHK53-BYP6744 [48] |
| BS-TRP1-$P_{GAL}$ | BS-TRP1 | $P_{GAL}$ | HindIII | GAGGTCGACGGTATCGATAAGCTTGGCATTACCACCATATACAT, TGCAGGAATTCGATATCAAGCTATAGTTTTTTCTCCTTGACGTTAAAG | pRS405-pGAL1-10-tc-apta-amber-FPA-TGA-Ape1 [71] |
| $P_{TPI1}$-Dsred-MET15 | ClhN-MET15 | $P_{TPI1}$, Dsred | NheI, AscI | TGGGTTAATTAACATCGCTAGCATGCAGGACGTTATGAAGAA, TCGCTTATTTAGAAGTGGCGCGCCCTATAAGAACAGGTGGTGGCG | TRT |
| $P_{ATG18}$-KanMX | UG6 | $P_{ATG18}$ | SalI | AGCTTCGTACGCTGCAGGTCGACCTGTAACCTTCTTGTTCGTG, | Genomic DNA |

| | | | | | |
|---|---|---|---|---|---|
| | | | | GAAGTTATATTAAGGGTTGTCGACTGTCAGGCCGAAGAAGAAAA | |
| BS-ZJLEU-$P_{TEF1}$-HFL1-GFP | BS-ZJLEU | $P_{TEF1}$-HFL1-GFP | ApaI, SpeI | CGAGGTCGACGGTATCGATACCACACACCATAGCTTCAAA, GGCGGCCGCTCTAGAACTAGTCTATTTGTATAGTTCATCCA | $P_{TEF1}$-HFL1-GFP-LEU |
| BS-ZJLEU-$P_{TEF1}$-HFL1-W371A-GFP | BS-ZJLEU | $P_{TEF1}$-HFL1-GFP | ApaI, SpeI | CGAGGTCGACGGTATCGATACCACACACCATAGCTTCAAA, GGCGGCCGCTCTAGAACTAGTCTATTTGTATAGTTCATCCA | $P_{TEF1}$-HFL1-W371A-GFP-LEU |
| BS-ZJLEU-$P_{TEF1}$-HFL1-I375A-GFP | BS-ZJLEU | $P_{TEF1}$-HFL1-GFP | ApaI, SpeI | CGAGGTCGACGGTATCGATACCACACACCATAGCTTCAAA, GGCGGCCGCTCTAGAACTAGTCTATTTGTATAGTTCATCCA | $P_{TEF1}$-HFL1-I375A-GFP-LEU |
| BS-ZJLEU-$P_{TEF1}$-HFL1-D384A-GFP | BS-ZJLEU | $P_{TEF1}$-HFL1-GFP | ApaI, SpeI | CGAGGTCGACGGTATCGATACCACACACCATAGCTTCAAA, GGCGGCCGCTCTAGAACTAGTCTATTTGTATAGTTCATCCA | $P_{TEF1}$-HFL1-D384A-GFP-LEU |
| BS-ZJLEU-$P_{TEF1}$-HFL1-Y387A-GFP | BS-ZJLEU | $P_{TEF1}$-HFL1-GFP | ApaI, SpeI | CGAGGTCGACGGTATCGATACCACACACCATAGCTTCAAA, GGCGGCCGCTCTAGAACTAGTCTATTTGTATAGTTCATCCA | $P_{TEF1}$-HFL1-Y387A-GFP-LEU |
| BS-ZJLEU-$P_{TEF1}$-HFL1-W371AI375A-GFP | BS-ZJLEU | $P_{TEF1}$-HFL1-GFP | ApaI, SpeI | CGAGGTCGACGGTATCGATACCACACACCATAGCTTCAAA, GGCGGCCGCTCTAGAACTAGTCTATTTGTATAGTTCATCCA | $P_{TEF1}$-HFL1-W371AI375A-GFP-LEU |
| BS-ZJLEU-$P_{TEF1}$-HFL1-D384AY387A-GFP | BS-ZJLEU | $P_{TEF1}$-HFL1-GFP | ApaI, SpeI | CGAGGTCGACGGTATCGATACCACACACCATAGCTTCAAA, GGCGGCCGCTCTAGAACTAGTCTATTTGTATAGTTCATCCA | $P_{TEF1}$-HFL1-D384AY387A-GFP-LEU |
| BS-ZJLEU-$P_{TEF1}$-HFL1-W371AI375AD384AY387A-GFP | BS-ZJLEU | $P_{TEF1}$-HFL1-GFP | ApaI, SpeI | CGAGGTCGACGGTATCGATACCACACACCATAGCTTCAAA, GGCGGCCGCTCTAGAACTAGTCTATTTGTATAGTTCATCCA | $P_{TEF1}$-HFL1-W371AI375AD384AY387A-GFP-LEU |

| | | | | | |
|---|---|---|---|---|---|
| $P_{CUP1}$-GFP-Vma13-Ura | ClhN-GFP | $P_{CUP1}$; Vma13 | AscI | CGTGGGATTCCTGGGCTAGTTAGAAAAAGACATTTTTGCTGTCAGTCAC, AGCGATGTTAATTAACATGATGACTTCTATATGATATTGCACTAACAAGAAGATATTATAATGCA; CTATACAAACCTAGGATGGGCGCAACCAAAATTTTAATGGAC, CGCTTATTTAGAAGTGGCGCGCCTTATTTGAAGGTATATCCAATGATTGCCTGC | Genomic DNA; $P_{CUP1}$-GFP-Pho8-406 |
| PCUP1-Vac8-GFP-Ura | ClhN-GFP | $P_{CUP1}$; Vac8 | AscI | CGTGGGATTCCTGGGCTAGTTAGAAAAAGACATTTTTGCTGTCAGTCAC, ACAACATGAACCCATGATGACTTCTATATGATATTGCACTAACAAGAAGATATTATAATGCA; CATATAGAAGTCATCATGGGTTCATGTTGTAGTTGCTTGAAAG, AGCGATGTTAATTAAATGTAAAAATTGTAAAATCTGTTGAGTAATATTATACAATTCCAAACTTG | Genomic DNA; $P_{CUP1}$-GFP-Pho8-406 |
| BS-Trp-$P_{CUP1}$-4myc-Pho8 | BS-Trp-$P_{CUP1}$-4myc-Ub | Pho8 | Avr II, Sac II | CTGAAGAAGATTTGCCTAGGATGATGACTCACACATTACCAAGCGAAC, CAGTAATAACCCACCGCGGGAGAGTTAGATAGGATCAGTTGGTCAACTC | Genomic DNA |
| $P_{CUP1}$-4V5-Pho8-Met | $P_{CUP1}$-4V5-Ub-Met | Pho8 | Avr II, Asc I | GTTTGGATTCTACTCCTAGGATGATGACTCACACATTACCAAGCGAAC, CGCTTATTTAGAAGTGGCGCGCCGAGAGTTAGATAGGATCAGTTGGTCAACTC | Genomic DNA |
| Tgl3-GFP [72] | (N/A) | (N/A) | (N/A) | (N/A) | (N/A) |
| BS-URA3-GFP-Atg8 [67] | (N/A) | (N/A) | (N/A) | (N/A) | (N/A) |
| BS-URA3-Atg18-2GFP [67] | (N/A) | (N/A) | (N/A) | (N/A) | (N/A) |

## Table S2. Plasmids Set II#.

#Fragments from parental plasmids were obtained by PCR amplification.

| Plasmid | Primers for Plasmid Backbone | Templates /Sources | Primers for Inserted Fragment | Templates /Sources |
|---|---|---|---|---|
| BS-TRP1-$P_{GAL}$-GFP-Cps1 | GCTTGATATCGAATTCCTGCA, TATAGTTTTTTCTCCTTGACGTTAAAGTATAGAGG | BS-TRP1-$P_{GAL}$ | TTAACGTCAAGGAGAAAAAACTATAATGTCTAAAGGTGAAGAATTATTC, GGGCTGCAGGAATTCGATATCAAGCCTTTACGCAAGTGGTTTTGA | $P_{CUP1}$-GFP-Cps1-406 |
| BS-TRP1-TetO7-mRuby3-Pho8 | GGAGCCGGAACAAGTGACAT, GCCTGCTGGATGAATAGCTT | BS-TRP1-TetO7-GFP-Pho8 | AAGCTATTCATCCAGCAGGC, CTCTTCGCCCTTAGACACCATCGCCGCTATCGATGTTAACA | BS-TRP1-TetO7-GFP-Pho8 |
| | | | ATGGTGTCTAAGGGCGAAGA, TCATGTCACTTGTTCCGGCTCCCTTGTACAGCTCGTCCATGC | NCS-mRuby3[73] |
| P1K-3HA-Atg8-MET15 | TGAGGCGCGCCACTTCTA, CCTCAACCTTTAATGGTTCC | $P_{ATG8}$-MET15 | GGAACCATTAAAGGTTGAGGAGGGGATTGATAAGAGAATCT, TTTAGAAGTGGCGCGCCTCACCTGCCAAATGTATTTTCTC | 3HA-ATG8-306 |
| P1K-3HA-Atg8ΔGR-MET15 | TGAGGCGCGCCACTTCTA, CCTCAACCTTTAATGGTTCC | $P_{ATG8}$-MET15 | GGAACCATTAAAGGTTGAGGAGGGGATTGATAAGAGAATCT, TTTAGAAGTGGCGCGCCTCAAAATGTATTTTCTCCTGAGTAAGT | 3HA-ATG8-306 |
| P1K-3HA-Atg8ΔR-MET15 | TGAGGCGCGCCACTTCTA, CCTCAACCTTTAATGGTTCC | $P_{ATG8}$-MET15 | GGAACCATTAAAGGTTGAGGAGGGGATTGATAAGAGAATCT, TTTAGAAGTGGCGCGCCTCAGCCAAATGTATTTTCTCCTG | 3HA-ATG8-306 |
| ClhN-flag-HIS5(S.p.) | GATAAACCTAGGTGAGGCGCGCCACTTCTA, CTCACCTAGGTTTATCATCATCATCTTTATAATCGCTAGCGATGTTAATTAACC | ClhN-HIS5(S.p.) | (N/A) | (N/A) |

| | | | | |
|---|---|---|---|---|
| P1K-HFL1-8flag-LEU | GGCGCGCCACTTCTAAATAAGCGAATTTCTTATGATTTAT, AACCCAGGAATCCCACGACCACAAGCCATTGATGACCTAC | UG74-8flag | ATAAATCATAAGAAATTCGCTTATTTAGAAGTGGCGCGCC, GTAGGTCATCAATGGCTTGTGGTCGTGGGATTCCTGGGTT | P1K-HFL1-GFP-LEU |
| P1K-HFL1-W371A-8flag-LEU | GGCGCGCCACTTCTAAATAAGCGAATTTCTTATGATTTAT, AACCCAGGAATCCCACGACCACAAGCCATTGATGACCTAC | UG74-8flag | ATAAATCATAAGAAATTCGCTTATTTAGAAGTGGCGCGCC, GTAGGTCATCAATGGCTTGTGGTCGTGGGATTCCTGGGTT | P1K-HFL1-W371A-GFP-LEU |
| P1K-HFL1-I375A-8flag-LEU | GGCGCGCCACTTCTAAATAAGCGAATTTCTTATGATTTAT, AACCCAGGAATCCCACGACCACAAGCCATTGATGACCTAC | UG74-8flag | ATAAATCATAAGAAATTCGCTTATTTAGAAGTGGCGCGCC, GTAGGTCATCAATGGCTTGTGGTCGTGGGATTCCTGGGTT | P1K-HFL1-I375A-GFP-LEU |
| P1K-HFL1-D384A-8flag-LEU | GGCGCGCCACTTCTAAATAAGCGAATTTCTTATGATTTAT, AACCCAGGAATCCCACGACCACAAGCCATTGATGACCTAC | UG74-8flag | ATAAATCATAAGAAATTCGCTTATTTAGAAGTGGCGCGCC, GTAGGTCATCAATGGCTTGTGGTCGTGGGATTCCTGGGTT | P1K-HFL1-D384A-GFP-LEU |
| P1K-HFL1-Y387A-8flag-LEU | GGCGCGCCACTTCTAAATAAGCGAATTTCTTATGATTTAT, AACCCAGGAATCCCACGACCACAAGCCATTGATGACCTAC | UG74-8flag | ATAAATCATAAGAAATTCGCTTATTTAGAAGTGGCGCGCC, GTAGGTCATCAATGGCTTGTGGTCGTGGGATTCCTGGGTT | P1K-HFL1-Y387A-GFP-LEU |
| P1K-HFL1-W371AI375A8flag-LEU | GGCGCGCCACTTCTAAATAAGCGAATTTCTTATGATTTAT, AACCCAGGAATCCCACGACCACAAGCCATTGATGACCTAC | UG74-8flag | ATAAATCATAAGAAATTCGCTTATTTAGAAGTGGCGCGCC, GTAGGTCATCAATGGCTTGTGGTCGTGGGATTCCTGGGTT | P1K-HFL1-W371AI375A-GFP-LEU |
| P1K-HFL1-D384AY387A-8flag-LEU | GGCGCGCCACTTCTAAATAAGCGAATTTCTTATGATTTAT, AACCCAGGAATCCCACGACCACAAGCCATTGATGACCTAC | UG74-8flag | ATAAATCATAAGAAATTCGCTTATTTAGAAGTGGCGCGCC, GTAGGTCATCAATGGCTTGTGGTCGTGGGATTCCTGGGTT | P1K-HFL1-D384AY387A-GFP-LEU |

| | | | | |
|---|---|---|---|---|
| P1K-HFL1-W371AI375AD384AY387A-8flag-LEU | GGCGCGCCACTTCTAAATAAGCGAATTTCTTATGATTTAT, AACCCAGGAATCCCACGACCACAAGCCATTGATGACCTAC | UG74-8flag | ATAAATCATAAGAAATTCGCTTATTTAGAAGTGGCGCGCC, GTAGGTCATCAATGGCTTGTGGTCGTGGGATTCCTGGGTT | P1K-HFL1-W371AI375AD384AY387A-GFP-LEU |
| $P_{CUP1}$-mRuby3-Pho8 | GACGAGCTGTACAAGGGAGCCGGAACAAGTGACATGATG, GCCCTTAGACACCATGTTACTAGTCGATGACTTCTATATGATATTGCAC | $P_{CUP1}$-GFP-Pho8-406 | ATGGTGTCTAAGGGCGAAGAGCTG, CTTGTACAGCTCGTCCATGCCAC | BS-TRP1-TetO7-mRuby3-Pho8 |

**Table S3. Plasmids Set III[&].**

| & Point mutations constructed by site-directed mutagenesis. | | |
|---|---|---|
| Plasmid | Primers for site-directed mutagenesis | Templates /Sources |
| $P_{CUP1}$-GFP-PHO8(K(24,25,30,33)R) | AGAAGGAGACGGATCTCGAGGAGATCAAGGATAATAGTATCCACTGTG, CCTTGATCTCCTCGAGATCCGTCTCCTTCTAGGACGAGAG | $P_{CUP1}$-GFP-PHO8-406 |
| $P_{CUP1}$-GFP-PHO8(K(24,25,30)R) | AGAAGGAGACGGATCTCGAGGAGATCAAGGATAATAGTATCCACTGTG, CCTTGATCTCCTCGAGATCCGTCTCCTTCTAGGACGAGAG | $P_{CUP1}$-GFP-PHO8-406 |
| $P_{CUP1}$-GFP-PHO8(K(30,33)R) | AGAAGGAGACGGATCTCGAGGAGATCAAGGATAATAGTATCCACTGTG, CCTTGATCTCCTCGAGATCCGTCTCCTTCTAGGACGAGAG | $P_{CUP1}$-GFP-PHO8-406 |
| $P_{CUP1}$-GFP-PHO8(K(33)R) | AGAAGGAGACGGATCTCGAGGAGATCAAGGATAATAGTATCCACTGTG, CCTTGATCTCCTCGAGATCCGTCTCCTTCTAGGACGAGAG | $P_{CUP1}$-GFP-PHO8-406 |
| ClhN-P1K-3VSV-Atg8(T56A T87A)-MET15 | CTAGTTCCTGCTGACCTTGCGGTAGGGCAATTTG & GCAAGGTCAGCAGGAACTAGATATTTACGCTTATC, GATACTTTGCCACCTGCTGCGGCGTTGATG & CAGCAGGTGGCAAAGTATCATTGACAAAAATGAAG | ClhN-P1K-3VSV-Atg8-MET15 |
| ClhN-P1K-3VSV-Atg8(P85A P86A)-MET15 | CATTTTTGTCAATGATACTTTGGCCGCTACTGCGGCGTTG, GTATCATTGACAAAAATGAAGATGGCCTTCTCAGG | ClhN-P1K-3VSV-Atg8-MET15 |
| ClhN-P1K-3VSV-Atg8(P85L P86L)-MET15 | CATTTTTGTCAATGATACTTTGTTGTTGACTGCGGCGTTG, GTATCATTGACAAAAATGAAGATGGCCTTCTCAGG | ClhN-P1K-3VSV-Atg8-MET15 |
| ClhN-P1K-3VSV-Atg8(T56A R65A T87A)-MET15 | CCGTAGGGCAATTTGTTTATGTTATAGCGAAGAGAATTATGC, CATAAACAAATTGCCCTACGGCAAGGTCAGCAGG | ClhN-P1K-3VSV-Atg8(T56A T87A)-MET15 |
| ClhN-P1K-3VSV-Atg8(R65A P85A P86A)-MET15 | CCGTAGGGCAATTTGTTTATGTTATAGCGAAGAGAATTATGC, CATAAACAAATTGCCCTACGGTAAGGTCAGCAGG | ClhN-P1K-3VSV-Atg8(P85A P86A)-MET15 |
| ClhN-P1K-3VSV-Atg8(R65A P85L P86L)-MET15 | CCGTAGGGCAATTTGTTTATGTTATAGCGAAGAGAATTATGC, CATAAACAAATTGCCCTACGGTAAGGTCAGCAGG | ClhN-P1K-3VSV-Atg8(P85L P86L)-MET15 |
| ClhN-P1K-3VSV-Atg8(L55W T56A V61W R65A)-MET15 | AGGGCAATTTTGGTATGTTATAGCGAAGAGAATTATGCTACC, TAACATACCAAAATTGCCCTACCGCCCAGTCAGCAG | ClhN-P1K-3VSV-Atg8-MET15 |

**Table S4. Primers for gene knockout and tagging.**

| Operation | Primers |
|---|---|
| *ATG1* knockout | TCAAATCTCTTTTACAACACCAGACGAGAAATTAAGAAAGCTTCGTACGCTGCAGGTCG,<br>ATTTGTACTTAATAAGAAAACCATATTATGCATCACTTAGCATAGGCCACTAGTGGATC |
| *ATG2* knockout | AAATTAAGAGGAACCCTTTTTTTTTTTGATTTCGATACAGCTTCGTACGCTGCAGGTCG,<br>GAATTGAATATATATCAAAAATGTCTGCAAAAATTTTTAGCATAGGCCACTAGTGGATC |
| *ATG7* knockout | TCATTATATTTCAACAAATATAAGATAATCAAGAATAAAGCTTCGTACGCTGCAGGTCG,<br>ACCACAATATGTACCAATGCTATTATATGCAAAATATTAGCATAGGCCACTAGTGGATC |
| *ATG8* knockout | TAAAGTTGAGAAAATCATAATAAAATAATTACTAGAGACGCTTCGTACGCTGCAGGTCG,<br>TAGATGTTAACGCTTCATTTCTTTTCATATAAAAGACTAGCATAGGCCACTAGTGGATC |
| *ATG9* knockout | GAACAGCCTGAAATATCAAAATCACGGAATTATTAGGTTGCTTCGTACGCTGCAGGTCG,<br>TAGTTATATTGGATGATGTACACGACACAGTCTGCCTTAGCATAGGCCACTAGTGGATC |
| *ATG18* knockout | TCCAGTTAACTCTGTATCCTTTTCTTCTTCGGCCTGACAGCTTCGTACGCTGCAGGTCG,<br>GTGACGTACGGAAGGCAGCGCGAGACACTTCCGTGATCAGCATAGGCCACTAGTGGATC |
| *VAC8* knockout | GGGTGTTCTTTCTTCTGTACTATATATACATTTGCAACTGCTTCGTACGCTGCAGGTCG,<br>AAAAATTATAATGCCTAGTCCCGCTTTTGAAGAAAATCAGCATAGGCCACTAGTGGATC |
| *VID24* knockout | TCTTGTGCATGACACCAACACATATCGCAAGCTTGAGTCGCTTCGTACGCTGCAGGTCG,<br>AAAAGGGTATGCAGGTAAAAACGAATATATCACACATCAGCATAGGCCACTAGTGGATC |
| *VID28* knockout | TGCCCTCGTGTTAGATTGGTGATATATTACAGTTACACTGCTTCGTACGCTGCAGGTCG,<br>AAATTAATTGTAGGAAATACTACGAGTTCTGTGCATTCAGCATAGGCCACTAGTGGATC |
| *VTC1* knockout | TATCGAATACGATTAAACACTACGCCAGATTTCCACAATGCTTCGTACGCTGCAGGTCG,<br>TGCGTAACCCACGCTTACGATATTGGAATTACAATTTCAGCATAGGCCACTAGTGGATC |
| *VTC2* knockout | AAGAACGACTACACCTCAACATAACGACACTTTTTTGACGCTTCGTACGCTGCAGGTCG,<br>TAAAAACACATGGTCTCAGTAGATAGAGTACATATTCTAGCATAGGCCACTAGTGGATC |
| *VTC3* knockout | CGAACAGCAGAATTTGTCCTTGGTTTTCAGAGTTTGAAAGCTTCGTACGCTGCAGGTCG,<br>GTGTAATATATGTGTATATAAAAAATATACATGTTCTTAGCATAGGCCACTAGTGGATC |
| *VTC4* knockout | AATCGGCCAATAAAAGAGCATAACAAGGCAGGAACAGCTGCTTCGTACGCTGCAGGTCG,<br>TACTTAATTATACAGTAAAAAAAACACGCTGTGTATTCAGCATAGGCCACTAGTGGATC |

| | |
|---|---|
| *EGO1* knockout | TAGCGGTAGTGGATACAACGACAGATTTAAGTCGTAAAAGCTTCGTACGCTGCAGGTCG,<br>AAGCTTGTATACCAATGATGTTATACAAAAAGTTCATTAGCATAGGCCACTAGTGGATC |
| *GTR2* knockout | AACAAAACTCCAGGACAACGGTACTAATACACATACAACGCTTCGTACGCTGCAGGTCG,<br>ATCTATATACCCTAATATTTTCATGCCTTACGTCTTTCAGCATAGGCCACTAGTGGATC |
| *EGO3* knockout | GTTATCAGCAGCAAACGGTATCCAAAATATTGAAGCAATGCTTCGTACGCTGCAGGTCG,<br>GAAAAGGTGTGGCCTCGATACATATTGTTAAACCCATCAGCATAGGCCACTAGTGGATC |
| *VPS27* knockout | CTAAGGTGAATGAGTAGTGAGTAAAGAACTAAGAACAGTGCTTCGTACGCTGCAGGTCG,<br>GTTTCTTTTTACAAATACATAGAAAAGGCTACAATATTAGCATAGGCCACTAGTGGATC |
| *VPS23* knockout | TCTTAACGGCCAAGAAAAGAGAGAGAGTGAAGAGCAACGGCTTCGTACGCTGCAGGTCG,<br>TTTTTATGGCACTTCGGCGATGCGAAAGAAAGTGAGTCAGCATAGGCCACTAGTGGATC |
| *SNF7* knockout | CGGAAGCAGCAGAAACATAACAGTATTGATAAATAAGGCGCTTCGTACGCTGCAGGTCG,<br>TTTTTTTTTCTTTCATCTAAACCGCATAGAACACGTTCAGCATAGGCCACTAGTGGATC |
| *VPS36* knockout | GGAAGTGTGTTTTGAAAGTCATTCTTTTTTTTTCAAAAGGCTTCGTACGCTGCAGGTCG,<br>TTTTTTTTTCTTTCATCTAAACCGCATAGAACACGTTCAGCATAGGCCACTAGTGGATC |
| *VPS4* knockout | ATAAAGCAGCATAGAGTGCCTATAGTAGATGGGGTACAAGCTTCGTACGCTGCAGGTCG,<br>ACACAAGAAATCTACATTAGCACGTTAATCAATTGACTAGCATAGGCCACTAGTGGATC |
| *FBP1* tagging | GAAATTGACAAATTTTTAGACCATATTGGCAAGTCACAGGCTTCGTACGCTGCAGGTCG,<br>GAACAAAGAAAATAAGAAAAGAAGGCGATCATTGAACTAGCATAGGCCACTAGTGGATC |
| *VPS23* tagging | GTGCGATGGCACATCCAACGAATCACCTCACCGTTATCGGCTTCGTACGCTGCAGGTCG,<br>TTTTTATGGCACTTCGGCGATGCGAAAGAAAGTGAGTCAGCATAGGCCACTAGTGGATC |
| *PEP4* knockout | CCTAGTATTTAATCCAAATAAAATTCAAACAAAAACCAAAACTAACGCTTCGTACGCTGCAGGTCG,<br>GATGGCAGAAAAGGATAGGGCGGAGAAGTAAGAAAAGTTTAGCTCAGCATAGGCCACTAGTGGATC |
| *VPS4* tagging | CAAGAACAGTTCACTAGAGATTTTGGTCAAGAAGGTAACGCTTCGTACGCTGCAGGTCG,<br>ACACAAGAAATCTACATTAGCACGTTAATCAATTGACTAGCATAGGCCACTAGTGGATC |
| *ATG15* knockout | GCATTACAATTAAAGGAAACAAGGGAAATATTCTATTGAGCTTCGTACGCTGCAGGTCG,<br>TAAAACAACACTAGGGTCATAATAGATGTATGGGTCTTAGCATAGGCCACTAGTGGATC |
| *NCR1* knockout | CATCTCCAAAAAGAACAAGAGCAGAACTTCGCTTCGTACGCTGCAGGTCG,<br>ACAGATTTTCTGGTGTGGCATCAGGCATAGGCCACTAGTGGATC |
| *NPC2* knockout | TCCTTCAAAGCTAGCACGCCTTCGCTTCGTACGCTGCAGGTCG,<br>ACACGGATCAATGAGTTGTATGAATCAGGCATAGGCCACTAGTGGATC |

| | |
|---|---|
| *VBA4* tagging | AATATTCATTTTCAACAGAATCCATTGTCGTAGTCAAAATTGTTTGTCACTATCTGGTGACGGTGCTGGTTTAATTAACATGTC,<br>GAAAAAAGAAAAAAAGTATTTGTATTATTTAAAGTATACATTTTCATCTACCGGTATAGGCCACTAGTGGATCTGATATCATCG |
| *NCR1* tagging | ACTTTCATTGTTTGGTGGTGAAAGCTATAGGGACGATTCCATCGAAGCAGAAGATGGTGACGGTGCTGGTTTAATTAACATGTC,<br>TAACGAATTTTTACCTATTTTTTCACTACGTAAAATATAGTATAATCTGCTATGGATAGGCCACTAGTGGATCTGATATCATCG |
| *YPQ1* tagging | CGTCATATTTTCCCAGTTTTTCATTTACAAAAGAAATAAAAAATTTATACTGAATGGTGACGGTGCTGGTTTAATTAACATGTC,<br>AAAAAAAAAGTTTGTGAGGTCAAAAATATGTTAAATAAATATTAGATAGAACATGATAGGCCACTAGTGGATCTGATATCATCG |
| *YPL162C* tagging | CTCAAATGAAGTGCCTAACAAGGATAACAACATTTCCAGCTATGGTAGCATAATAGGTGACGGTGCTGGTTTAATTAACATGTC,<br>TCAATTAAGCAAGGAAAGGAAATAAACAGTGCATGCTTTCCTTATCCTTGGAATAATAGGCCACTAGTGGATCTGATATCATCG |
| *DPP1* tagging | CACCCATCAGAGGATCCCGGATGAGGAATTACATCCTTTGTCCGATGAAGGTATGGGTGACGGTGCTGGTTTAATTAACATGTC,<br>AACAATAAATACGTATATTTCGTATGTCATGTGGAGTATATATTCTTTTTTATTCATAGGCCACTAGTGGATCTGATATCATCG |
| *YCF1* tagging | TAACAAATCATTGTTCTATTCACTGTGCATGGAGGCTGGTTTGGTCAATGAAAATGGTGACGGTGCTGGTTTAATTAACATGTC,<br>TTTATCCATCCTACGTACCAGATTGTGCGGGACAGGTTTTTATTAGTTTCACAGTATAGGCCACTAGTGGATCTGATATCATCG |

**Table S5. Strains used in this study.**

| Name | Genotype |
|---|---|
| BY4741 [74] | *MATα his3Δ1 leu2Δ met15Δ ura3Δ* |
| DJ01 | BY4741 *MATα his3Δ1 leu2Δ met15Δ ura3Δ trp1Δ::natMX* |
| DY03 | BY4741 *MATα his3Δ1 leu2Δ met15Δ ura3Δ pho8Δ60 pho13Δ::LEU2* |
| YMJ83 | DJ01 $P_{URA3}$*:: URA3(K.l.)·TetR'-Ssn6 VPS23-IAA17-4flag::HIS5(S.p.)* $P_{ADH1}$*::* $P_{ADH1}$*-TIR1-9myc·MET15* $T_{TRP1}$*::TRP1(K.l.)·TetO7-GFP-PHO8* |
| YMJ89 | DJ01 $P_{URA3}$*:: URA3(K.l.)·TetR'-Ssn6* $P_{VAC8}$*-IAA17-4flag-VAM3::HIS5(S.p.)* $P_{ADH1}$*::* $P_{ADH1}$*-TIR1-9myc·MET15* $T_{TRP1}$*::TRP1(K.l.)·TetO7-GFP-PHO8* |
| YMJ90 | DJ01 $P_{URA3}$*:: URA3(K.l.)·TetR'-Ssn6* $P_{VAC8}$*-IAA17-4flag-VPS39::HIS5(S.p.)* $P_{ADH1}$*::* $P_{ADH1}$*-TIR1-9myc·MET15* $T_{TRP1}$*::TRP1(K.l.)·TetO7-GFP-PHO8* |
| YMJ98 | DJ01 *PHO8::*$P_{CUP1}$*-GFP-PHO8·URA3* |
| YMJ221 | DJ01 *VPH1-2×GFP::URA3(K.l.)* |
| YMJ222 | DJ01 *YPQ2-2×GFP::URA3(K.l.)* |
| YMJ801 | DJ01 *HIS3 PHO8::*$P_{CUP1}$*-GFP-PHO8·URA3 MET15* |
| YMJ802 | DJ01 *vps4Δ::HIS5(S.p.) PHO8::*$P_{CUP1}$*-GFP-PHO8·URA3 MET15* |
| YMJ803 | DJ01 *vps23Δ::HIS5(S.p.) PHO8::*$P_{CUP1}$*-GFP-PHO8·URA3 MET15* |
| YMJ804 | DJ01 *atg8Δ::MET15 PHO8::*$P_{CUP1}$*-GFP-PHO8·URA3 HIS3* |
| YMJ806 | DJ01 *vps4Δ::HIS5(S.p.) PHO8::*$P_{CUP1}$*-GFP-PHO8·URA3 atg8Δ::MET15* |
| YMJ807 | DJ01 *vps23Δ::HIS5(S.p.) PHO8::*$P_{CUP1}$*-GFP-PHO8·URA3 atg8Δ::MET15* |
| YMJ224 | DJ01 *3HA-ATG8ΔGR::MET15 PHO8::*$P_{CUP1}$*-GFP-PHO8·URA3 HIS3* |
| YMJ225 | DJ01 *3HA-ATG8ΔR::MET15 PHO8::*$P_{CUP1}$*-GFP-PHO8·URA3 atg4Δ::HIS5(S.p.)* |
| YMJ226 | DJ01 *3HA-ATG8::MET15 PHO8::*$P_{CUP1}$*-GFP-PHO8·URA3 HIS3* |
| YMJ729 | DJ01 *OSH1::*$P_{CUP1}$*-GFP-OSH1·URA3* |
| YMJ731 | DJ01 *vac8Δ:HIS5(S.p.) OSH1::*$P_{CUP1}$*-GFP-OSH1·URA3* |
| YMJ155 | DJ01 *FBP1-4V5::URA3(K.l.)* |
| YMJ156 | DJ01 *vid28Δ::HIS5(S.p.) FBP1-4V5::URA3(K.l.)* |
| YMJ157 | DJ01 *vid24Δ::HIS5(S.p.) FBP1-4V5::URA3(K.l.)* |
| YMJ111 | DY03 *atg1Δ::HIS5(S.p.) pho8Δ60::*$P_{CUP1}$*-GFP-PHO8::URA3* |

| | |
|---|---|
| YMJ112 | DY03 *atg2Δ::HIS5(S.p.) pho8Δ60::*$P_{CUP1}$*-GFP-PHO8::URA3* |
| YMJ114 | DY03 *atg7Δ::HIS5(S.p.) pho8Δ60::*$P_{CUP1}$*-GFP-PHO8::URA3* |
| YMJ115 | DY03 *atg8Δ::HIS5(S.p.) pho8Δ60::*$P_{CUP1}$*-GFP-PHO8::URA3* |
| YMJ116 | DY03 *atg9Δ::HIS5(S.p.) pho8Δ60::*$P_{CUP1}$*-GFP-PHO8::URA3* |
| YMJ117 | DY03 *atg18Δ::HIS5(S.p.) pho8Δ60::*$P_{CUP1}$*-GFP-PHO8::URA3* |
| YMJ118 | DJ01 *PHO8::*$P_{CUP1}$*-GFP-PHO8-URA3 HIS3* |
| YMJ119 | DY03 $P_{CUP1}$*-GFP-PHO8::URA3 HIS3* |
| YMJ100 | DJ01 *pep4Δ::HIS5(S.p.) PHO8::*$P_{CUP1}$*-GFP-PHO8·URA3* |
| YMJ101 | DJ01 *vps4Δ::HIS5(S.p.) PHO8::*$P_{CUP1}$*-GFP-PHO8·URA3* |
| YMJ102 | DJ01 *vps23Δ::HIS5(S.p.) PHO8::*$P_{CUP1}$*-GFP-PHO8·URA3* |
| YMJ103 | DJ01 *vps27Δ::HIS5(S.p.) PHO8::*$P_{CUP1}$*-GFP-PHO8·URA3* |
| YMJ104 | DJ01 *vps36Δ::HIS5(S.p.) PHO8::*$P_{CUP1}$*-GFP-PHO8·URA3* |
| YMJ105 | DJ01 *snf7Δ::HIS5(S.p.) PHO8::*$P_{CUP1}$*-GFP-PHO8·URA3* |
| YMJ107 | DJ01 *vid24Δ::HIS5(S.p.) PHO8::*$P_{CUP1}$*-GFP-PHO8·URA3* |
| YMJ216 | DJ01 *vac8Δ::HIS5(S.p.) PHO8::*$P_{CUP1}$*-GFP-PHO8·URA3* |
| YMJ218 | DJ01 *vid28Δ::HIS5(S.p.) PHO8::*$P_{CUP1}$*-GFP-PHO8·URA3* |
| YMJ410 | DY03 *HIS3* |
| YMJ411 | DY03 *atg1Δ::HIS5(S.p.)* |
| YMJ412 | DY03 *atg2Δ::HIS5(S.p.)* |
| YMJ414 | DY03 *atg7Δ::HIS5(S.p.)* |
| YMJ415 | DY03 *atg8Δ::HIS5(S.p.)* |
| YMJ416 | DY03 *atg9Δ::HIS5(S.p.)* |
| YMJ417 | DY03 *atg18Δ::HIS5(S.p.)* |
| YMJ421 | DJ01 *CPS1::*$P_{CUP1}$*-GFP-CPS1·URA3* |
| YMJ431 | DJ01 *vps4Δ::HIS5(S.p.) CPS1::*$P_{CUP1}$*-GFP-CPS1·URA3* |
| YMJ432 | DJ01 *vps23Δ::HIS5(S.p.) CPS1::*$P_{CUP1}$*-GFP-CPS1·URA3* |
| YMJ433 | DJ01 *vps27Δ::HIS5(S.p.) CPS1::*$P_{CUP1}$*-GFP-CPS1·URA3* |
| YMJ434 | DJ01 *vps36Δ::HIS5(S.p.) CPS1::*$P_{CUP1}$*-GFP-CPS1·URA3* |
| YMJ435 | DJ01 *snf7Δ::HIS5(S.p.) CPS1::*$P_{CUP1}$*-GFP-CPS1·URA3* |

| | |
|---|---|
| YMJ702 | DJ01 *TRP1(K.l.) URA3(K.l.)* |
| YMJ704 | DJ01 *pho8Δ::TRP1(K.l.) URA3(K.l.)* |
| YMJ705 | DJ01 *pho8Δ::*$P_{CUP1}$*-GFP-PHO8·URA3·TRP1(K.l.)* |
| YMJ720 | DJ01 *vtc1Δ:HIS5(S.p.) PHO8::*$P_{CUP1}$*-GFP-PHO8·URA3* |
| YMJ721 | DJ01 *vtc2Δ:HIS5(S.p.) PHO8::*$P_{CUP1}$*-GFP-PHO8·URA3* |
| YMJ722 | DJ01 *vtc3Δ:HIS5(S.p.) PHO8::*$P_{CUP1}$*-GFP-PHO8·URA3* |
| YMJ723 | DJ01 *vtc4Δ:HIS5(S.p.) PHO8::*$P_{CUP1}$*-GFP-PHO8·URA3* |
| YMJ724 | DJ01 *ego1Δ:HIS5(S.p.) PHO8::*$P_{CUP1}$*-GFP-PHO8·URA3* |
| YMJ725 | DJ01 *gtr2Δ:HIS5(S.p.) PHO8::*$P_{CUP1}$*-GFP-PHO8·URA3* |
| YMJ726 | DJ01 *ego3Δ:HIS5(S.p.) PHO8::*$P_{CUP1}$*-GFP-PHO8·URA3* |
| YMJ728 | DJ01 *atg15Δ:HIS5(S.p.) PHO8::*$P_{CUP1}$*-GFP-PHO8·URA3* |
| YMJ735 | DJ01 $P_{ADH1}$*::*$P_{ADH1}$*-TIR1-9myc·MET VPS23-IAA17-4flag::HIS5(S.p.)* $T_{TRP1}$*::TRP1(K.l.)·*$P_{GAL}$*-GFP-CPS1* |
| YMJ764 | DJ01 *HIS3 CPS1::*$P_{CUP1}$*-GFP-CPS1·URA3* |
| YMJ766 | DJ01 *VPS23-IAA17-4flag::HIS5(S.p.) CPS1::*$P_{CUP1}$*-GFP-CPS1·URA3* |
| YMJ770 | DJ01 *MET15 CPS1::*$P_{CUP1}$*-GFP-CPS1·URA3* |
| YMJ771 | DJ01 *atg8Δ::MET15 CPS1::*$P_{CUP1}$*-GFP-CPS1·URA3* |
| YCF030 | DJ01 *pho8Δ::TRP1(K.l.)* $Ter_{PHO8}$*::*$P_{CUP1}$*-GFP-PHO8(K(24,25,30,33)R)·URA(K.l.)* |
| YCF031 | DJ01 *pho8Δ::TRP1(K.l.)* $Ter_{PHO8}$*::*$P_{CUP1}$*-GFP-PHO8(K33R)·URA(K.l.)* |
| YCF032 | DJ01 *pho8Δ::TRP1(K.l.)* $Ter_{PHO8}$*::*$P_{CUP1}$*-GFP-PHO8(K(30,33)R)·URA(K.l.)* |
| YCF036 | DJ01 *pho8Δ::TRP1(K.l.)* $Ter_{PHO8}$*::*$P_{CUP1}$*-GFP-PHO8(K(24,25,30)R)·URA(K.l.)* |
| YCF037 | DJ01 *LEU(K.l.) MET15 PHO8::*$P_{CUP1}$*-GFP-PHO8·URA3(K.l.)* |
| YCF038 | DJ01 *LEU(K.l.) MET15 TLG3::TGL3-GFP* |
| YCF114 | DJ01 *ncr1Δ::LEU(K.l.) npc2Δ::MET15* |
| YCF117 | YCF114 *PHO8::*$P_{CUP1}$*-GFP-PHO8·URA3(K.l.)* |
| YCF120 | YCF114 *TLG3::TGL3-GFP* |
| YCF130 | YMJ115 $P_{ATG8}$*::*$P_{ATG8}$*-3VSV-ATG8·MET15* |
| YCF134 | YMJ115 $P_{ATG8}$*::*$P_{ATG8}$*-3VSV-ATG8(T56A T87A)·MET15* |
| YCF135 | YMJ115 $P_{ATG8}$*::*$P_{ATG8}$*-3VSV-ATG8(P85A P86A)·MET15* |

| | |
|---|---|
| YCF136 | YMJ115 $P_{ATG8}$::$P_{ATG8}$*-3VSV-ATG8(P85L P86L)·MET15* |
| YCF137 | YMJ115 $P_{ATG8}$::$P_{ATG8}$*-3VSV-ATG8(R65A P85A P86A)·MET15* |
| YCF138 | YMJ115 $P_{ATG8}$::$P_{ATG8}$*-3VSV-ATG8(R65A P85L P86L)·MET15* |
| YCF142 | YMJ115 $P_{ATG8}$::$P_{ATG8}$*-3VSV-ATG8(T56A R65A T87A)·MET15* |
| YCF151 | YMJ115 $P_{ATG8}$::$P_{ATG8}$*-3VSV-ATG8(L55W T56A V61W R65A)·MET15* |
| YMJ745 | DJ01 *hfl1Δ::HIS5(S.p.)* $P_{CUP1}$*-GFP-PHO8::URA3* |
| YCH437 | BY4741 *trp1Δ::natMX6 atg4Δ::HIS5 hfl1Δ::TRP1(k.l.) 3HA-ATG8ΔR::MET15 PHO8::*$P_{CUP1}$*-GFP-PHO8·URA3(K.l.)* |
| YCH601 | BY4741 *hfl1Δ::HIS3 PHO8::*$P_{CUP1}$*-GFP-PHO8·URA3(K.l.) LEU2::*$P_{HFL1}$*-HFL1-8flag* |
| YCH602 | BY4741 *hfl1Δ::HIS3 PHO8::*$P_{CUP1}$*-GFP-PHO8·URA3(K.l.) LEU2::*$P_{HFL1}$*-HFL1(W371A)-8flag* |
| YCH603 | BY4741 *hfl1Δ::HIS3 PHO8::*$P_{CUP1}$*-GFP-PHO8·URA3(K.l.) LEU2::*$P_{HFL1}$*-HFL1(I375A)-8flag* |
| YCH604 | BY4741 *hfl1Δ::HIS3 PHO8::*$P_{CUP1}$*-GFP-PHO8·URA3(K.l.) LEU2::*$P_{HFL1}$*-HFL1(D384A)-8flag* |
| YCH605 | BY4741 *hfl1Δ::HIS3 PHO8::*$P_{CUP1}$*-GFP-PHO8·URA3(K.l.) LEU2::*$P_{HFL1}$*-HFL1(Y387A)-8flag* |
| YCH606 | BY4741 *hfl1Δ::HIS3 PHO8::*$P_{CUP1}$*-GFP-PHO8·URA3(K.l.) LEU2::*$P_{HFL1}$*-HFL1(W371AI375A)-8flag* |
| YCH607 | BY4741 *hfl1Δ::HIS3 PHO8::*$P_{CUP1}$*-GFP-PHO8·URA3(K.l.) LEU2::*$P_{HFL1}$*-HFL1(D384AY387A)-8flag* |
| YCH608 | BY4741 *hfl1Δ::HIS3 PHO8::*$P_{CUP1}$*-GFP-PHO8·URA3(K.l.) LEU2::*$P_{HFL1}$*-HFL1(WIDY)-8flag* |
| YCH630 | BY4741 *atg8Δ::HIS3* $P_{ATG8}$::$P_{ATG8}$*-8V5-ATG8·MET17* |
| YCH631 | YCH630 *LEU2::*$P_{TEF1}$*-HFL1-GFP* |
| YCH632 | YCH630 *LEU2::*$P_{TEF1}$*-HFL1(W371A)-GFP* |
| YCH633 | YCH630 *LEU2::*$P_{TEF1}$*-HFL1(I375A)-GFP* |
| YCH634 | YCH630 *LEU2::*$P_{TEF1}$*-HFL1(D384A)-GFP* |
| YCH635 | YCH630 *LEU2::*$P_{TEF1}$*-HFL1(Y387A)-GFP* |
| YCH636 | YCH630 *LEU2::*$P_{TEF1}$*-HFL1(W371AI375A)-GFP* |
| YCH637 | YCH630 *LEU2::*$P_{TEF1}$*-HFL1(D384AY387A)-GFP* |
| YCH638 | YCH630 *LEU2::*$P_{TEF1}$*-HFL1(WIDY)-GFP* |
| YCH641 | BY4741 *LEU2::*$P_{TEF1}$*-HFL1-GFP::LEU2* $P_{ATG8}$:: $P_{ATG8}$*-3VSV-ATG8·MET17* |
| YCH642 | BY4741 *atg8Δ::HIS3 LEU2::*$P_{TEF1}$*-HFL1-GFP* $P_{ATG8}$::$P_{ATG8}$*-3VSV-ATG8·MET17* |
| YCH643 | BY4741 *atg8Δ::HIS3 LEU2::*$P_{TEF1}$*-HFL1-GFP* $P_{ATG8}$::$P_{ATG8}$*-3VSV-ATG8(T56AT87A)·MET17* |

| | |
|---|---|
| YCH644 | BY4741 atg8Δ::HIS3 LEU2::$P_{TEF1}$-HFL1-GFP $P_{ATG8}$::$P_{ATG8}$-3VSV-ATG8(P85AP86A)·MET17 |
| YCH645 | BY4741 atg8Δ::HIS3 LEU2::$P_{TEF1}$-HFL1-GFP $P_{ATG8}$::$P_{ATG8}$-3VSV-ATG8(P85LP86L)·MET17 |
| YCH646 | BY4741 atg8Δ::HIS3 LEU2::$P_{TEF1}$-HFL1-GFP $P_{ATG8}$::$P_{ATG8}$-3VSV-ATG8(R65AT56AT87A)·MET17 |
| YCH647 | BY4741 atg8Δ::HIS3 LEU2::$P_{TEF1}$-HFL1-GFP $P_{ATG8}$::$P_{ATG8}$-3VSV-ATG8(R65AP85AP86A)·MET17 |
| YCH648 | BY4741 atg8Δ::HIS3 LEU2::$P_{TEF1}$-HFL1-GFP $P_{ATG8}$::$P_{ATG8}$-3VSV-ATG8(R65AP85LP86L)·MET17 |
| YCH649 | BY4741 atg8Δ::HIS3 LEU2::$P_{TEF1}$-HFL1-GFP $P_{ATG8}$::$P_{ATG8}$-3VSV-ATG8(L55WT56AV61WR65A)·MET17 |
| YMJ211 | DJ01 EGO3-GFP·URA3(K.l.) |
| YMJ301 | DJ01 vps4Δ::HIS5(S.p.) VPH1-2×GFP·URA3(K.l.) |
| YMJ302 | DJ01 vps23Δ::HIS5(S.p.) VPH1-2×GFP·URA3(K.l.) |
| YMJ303 | DJ01 vps27Δ::HIS5(S.p.) VPH1-2×GFP·URA3(K.l.) |
| YMJ304 | DJ01 vps36Δ::HIS5(S.p.) VPH1-2×GFP·URA3(K.l.) |
| YMJ305 | DJ01 snf7Δ::HIS5(S.p.) VPH1-2×GFP·URA3(K.l.) |
| YMJ309 | DJ01 VPH1-2×GFP·URA3(K.l.) |
| YMJ310 | DY03 VPH1-2×GFP·URA3(K.l.) |
| YMJ311 | DY03 atg1Δ::HIS5(S.p.) VPH1-2×GFP·URA3(K.l.) |
| YMJ312 | DY03 atg2Δ::HIS5(S.p.) VPH1-2×GFP·URA3(K.l.) |
| YMJ314 | DY03 atg7Δ::HIS5(S.p.) VPH1-2×GFP·URA3(K.l.) |
| YMJ315 | DY03 atg8Δ::HIS5(S.p.) VPH1-2×GFP·URA3(K.l.) |
| YMJ316 | DY03 atg9Δ::HIS5(S.p.) VPH1-2×GFP·URA3(K.l.) |
| YMJ317 | DY03 atg18Δ::HIS5(S.p.) VPH1-2×GFP·URA3(K.l.) |
| YCF155 | TN124 metΔ hisΔ uraΔ atg8Δ::NAT |
| YCF156 | YCF155 $P_{ATG8}$::$P_{ATG8}$-3VSV-ATG8·MET |
| YCF157 | YCF155 $P_{ATG8}$::$P_{ATG8}$-3VSV-ATG8(T56A T87A)·MET |
| YCF158 | YCF155 $P_{ATG8}$::$P_{ATG8}$-3VSV-ATG8(P85A P86A)·MET |
| YCF159 | YCF155 $P_{ATG8}$::$P_{ATG8}$-3VSV-ATG8(P85L P86L)·MET |
| YCF165 | YCF155 $P_{ATG8}$::$P_{ATG8}$-3VSV-ATG8(T56A R65A T87A)·MET |
| YCF190 | DJ01 $P_{CUP1}$-4myc-PHO8·TRP(K.l.) |

| | |
|---|---|
| YCF193 | DJ01 *$P_{CUP1}$-4V5-PHO8·MET* |
| YCF732 | DJ01 *ATG18-2GFP·URA3(K.l.)* |
| YCF908 | DJ01 *$Ter_{ADH1}$::$P_{CUP1}$-VAC8-GFP·URA3(K.l.)* |
| YCF909 | DJ01 *$Ter_{ADH1}$::$P_{CUP1}$-GFP-VMA13·URA3(K.l.)* |
| YCF910 | DJ01 *$Ter_{ADH1}$::$P_{CUP1}$-mRuby3-PHO8·URA3(K.l.)* |
| YCF919 | DJ01 *VBA4-2GFP·URA3(K.l.)* |
| YCF920 | DJ01 *NCR1-2GFP·URA3(K.l.)* |
| YCF921 | DJ01 *YPQ1-2GFP·URA3(K.l.)* |
| YCF922 | DJ01 *YPL162C-2GFP·URA3(K.l.)* |
| YCF923 | DJ01 *DPP1-2GFP·URA3(K.l.)* |
| YCF924 | DJ01 *YCF1-2GFP·URA3(K.l.)* |
| YCH660 | DJ01 *LEU2::PTEF1-HFL1(WIDY)-GFP* |
| YCH661 | DJ01 *vps23Δ::HIS5(S.p.)  LEU2::PTEF1-HFL1-GFP* |
| YCH662 | DJ01 *atg8Δ::HIS5(S.p.)  LEU2::PTEF1-HFL1-GFP* |
| YCH663 | DJ01 *hfl1Δ::HIS5(S.p.) atg8△::natMX PHO8::$P_{CUP1}$-GFP-PHO8·URA3* |
| YCH664 | DY03 *atg4Δ::HIS5(S.p.) ATG8::GFP-ATG8△R·URA3 HFL1::$P_{HFL1}$-HFL1-4flag·natMX6* |
| YCH665 | DY03 *ATG8::GFP-ATG8·URA3 HFL1::$P_{HFL1}$-HFL1-4flag·natMX6* |
| YCH666 | DY03 *atg4Δ::HIS5(S.p.) ATG8::GFP-ATG8△R·URA3 HFL1::$P_{TEF1}$-HFL1-4flag·natMX6* |
| YCH667 | DY03 *ATG8::GFP-ATG8·URA3  HFL1::$P_{TEF1}$-HFL1-4flag·natMX6* |
| YMJ320 | DJ01 *hfl1Δ::HIS5(S.p.) VPH1-2×GFP::URA3(K.l.)* |
