# Supplementary material for: Membrane recruitment of Atg8 by Hfl1 facilitates turnover of vacuolar membrane proteins in yeast cells approaching stationary phase
Source: BMC Biol. 2021 Jun 4;19:117. doi: 10.1186/s12915-021-01048-7 (PMC8176713; doi:10.1186/s12915-021-01048-7)

Fig. 1B

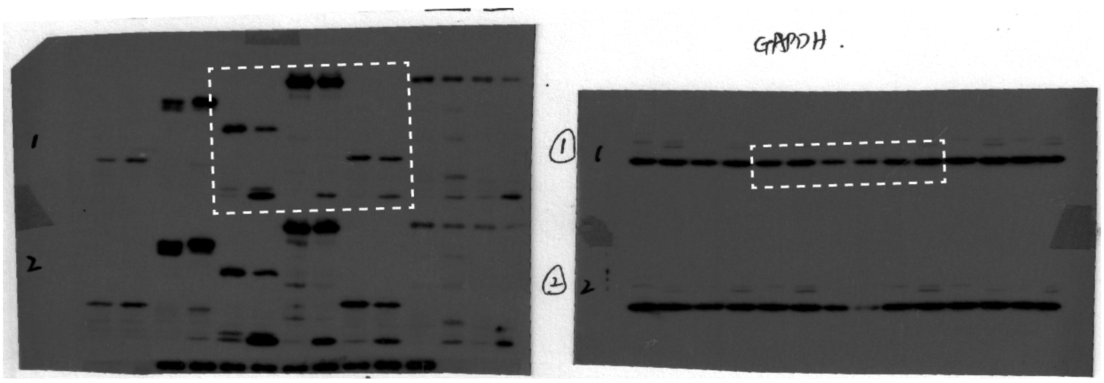

Fig. 1F

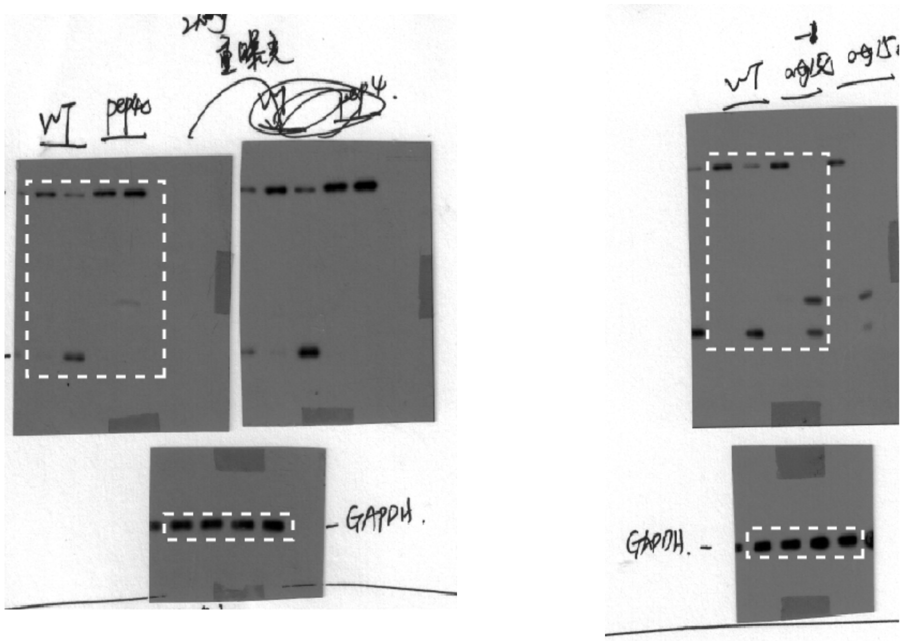

Fig. 3B

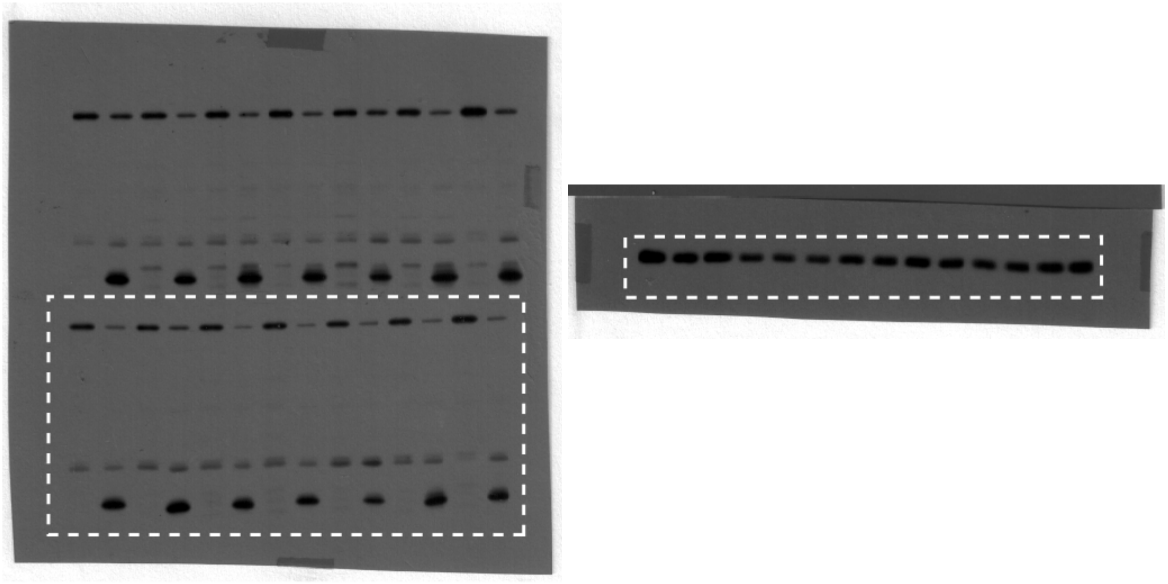

Fig. 3D

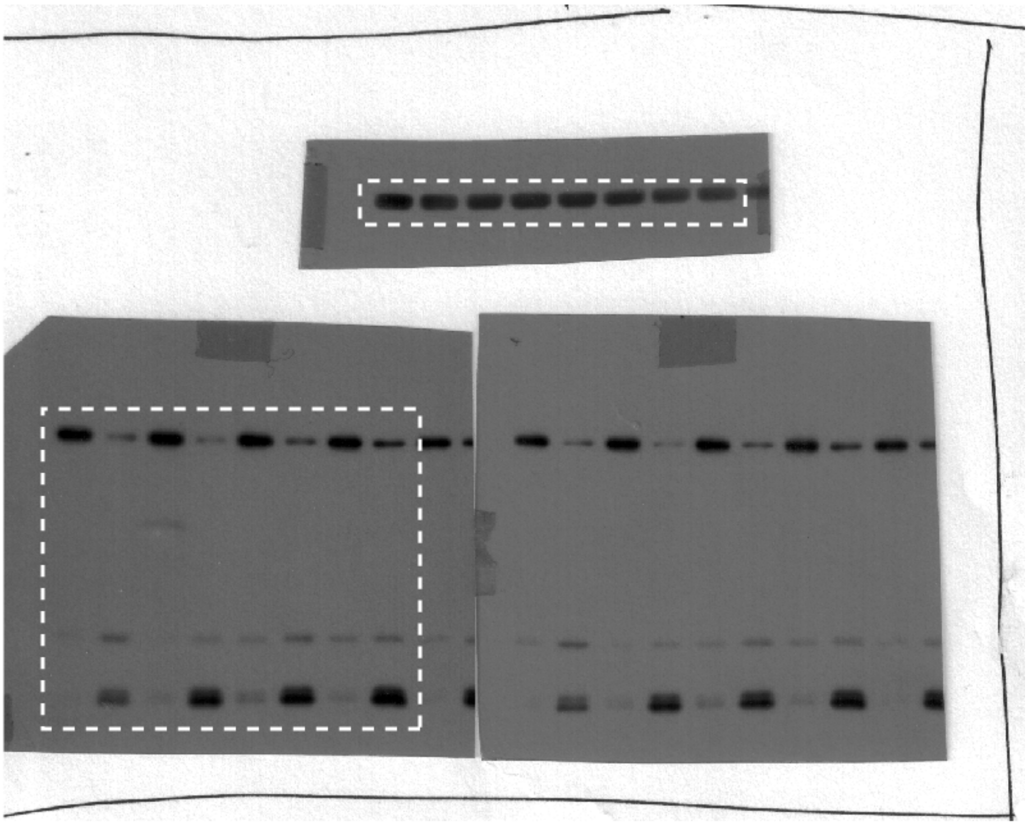

Fig. 4B

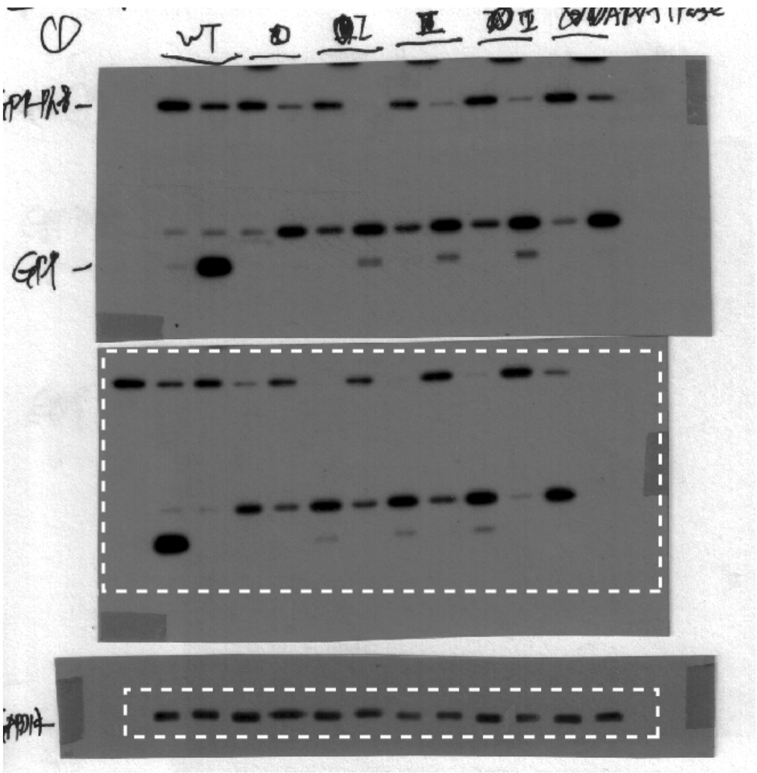

Fig. 4D

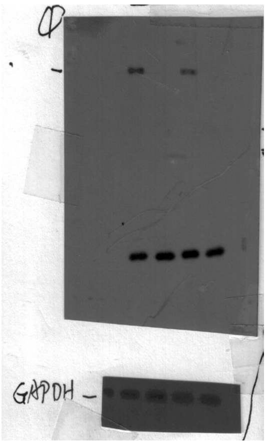

Fig. 4F

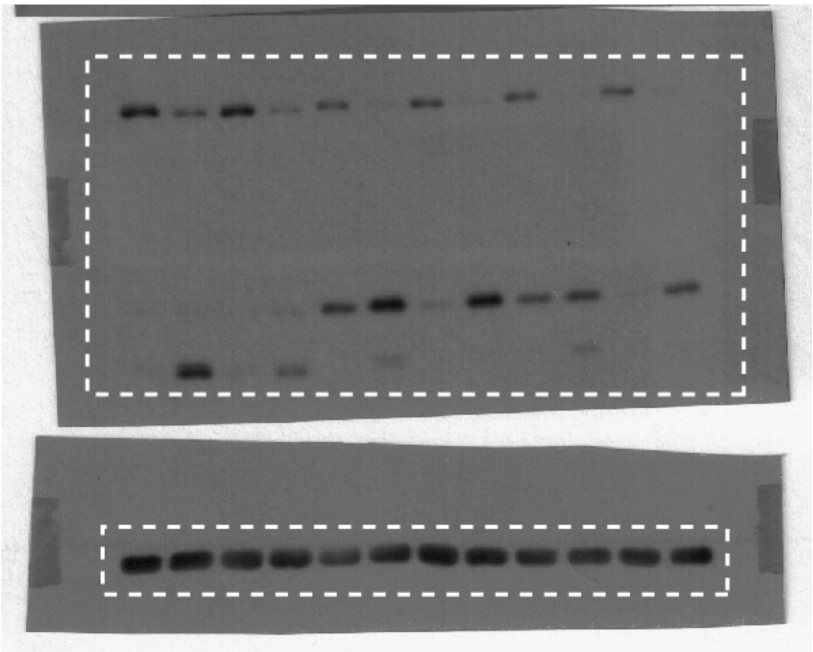

Fig. 5B

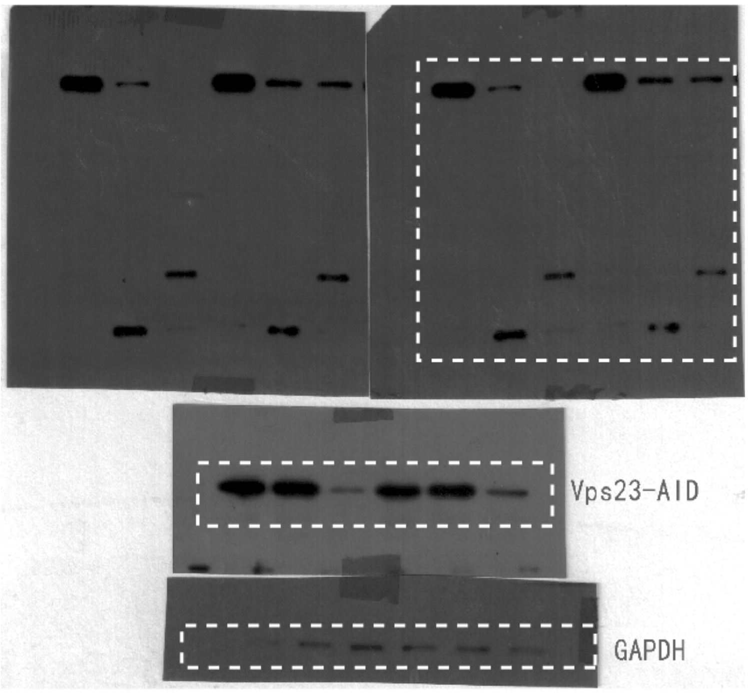

Fig. 5C

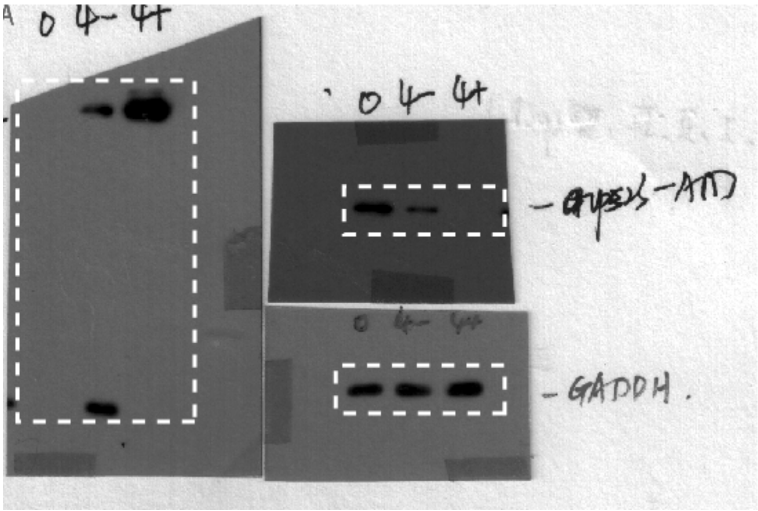

Fig. 6B

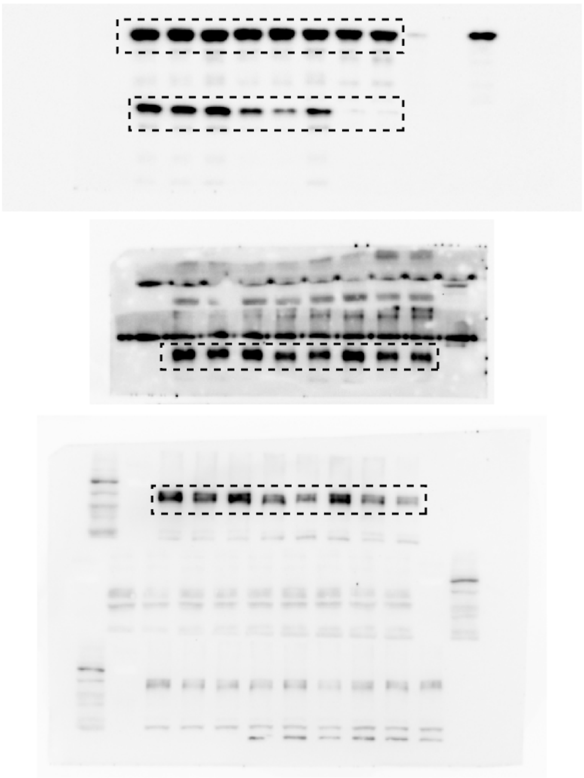

Fig. 6D

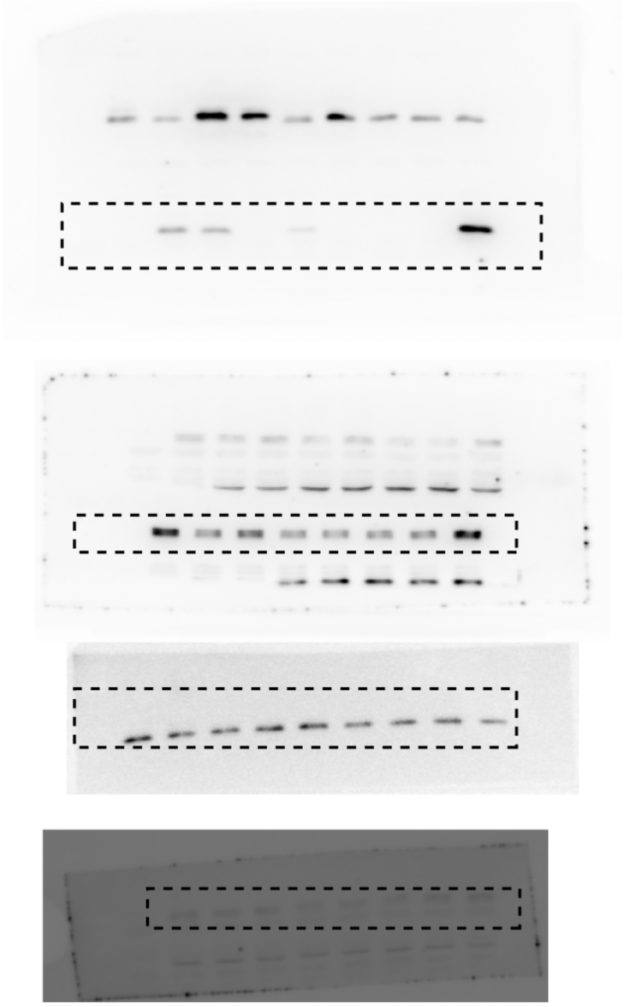

Fig. S1B

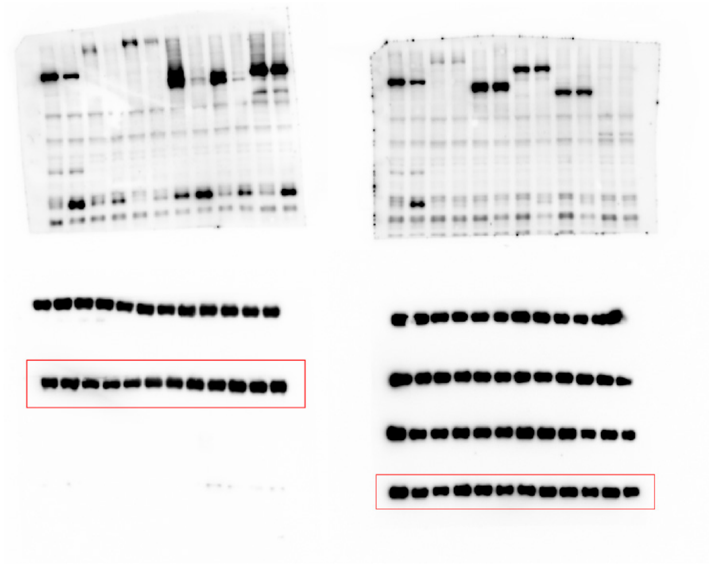

Fig. S1C

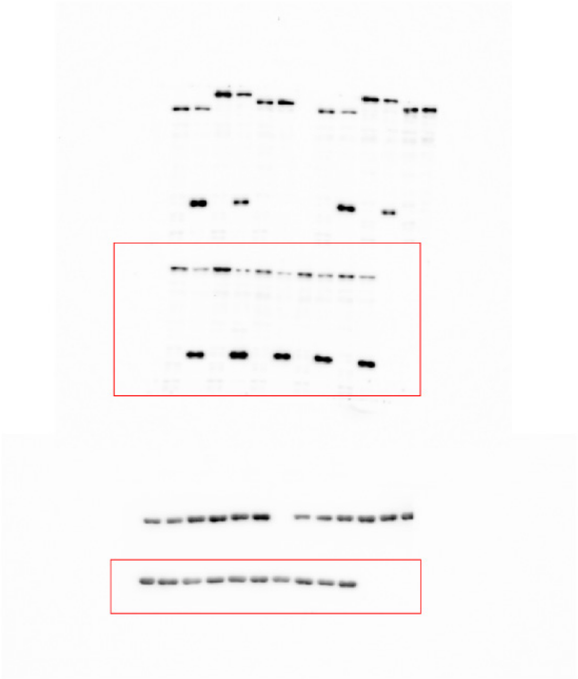

Fig. S1F

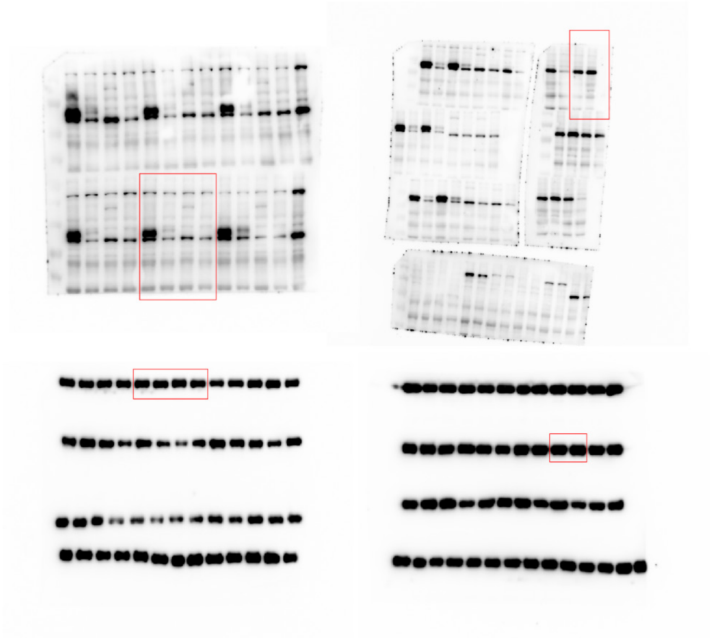

Fig. S2D

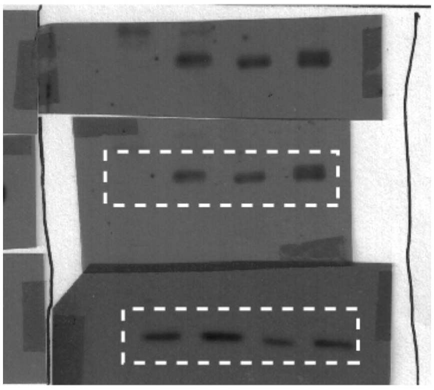

Fig. S3B

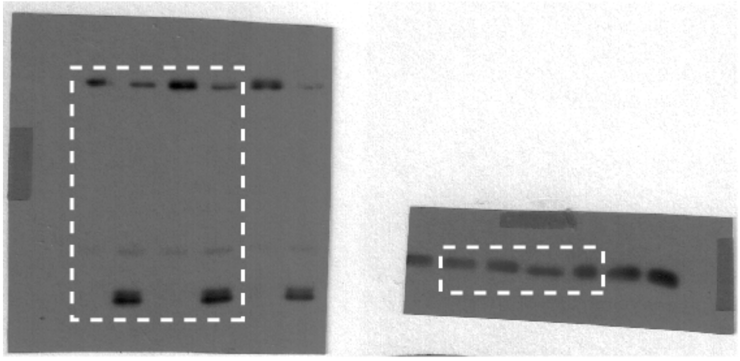

Fig. S3C

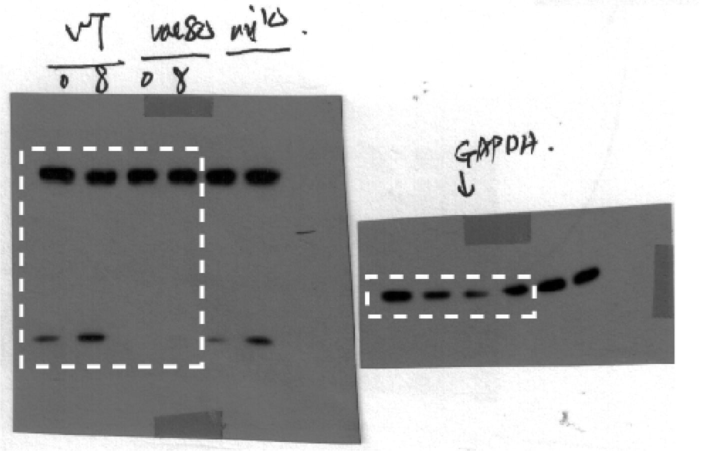

Fig. S3E

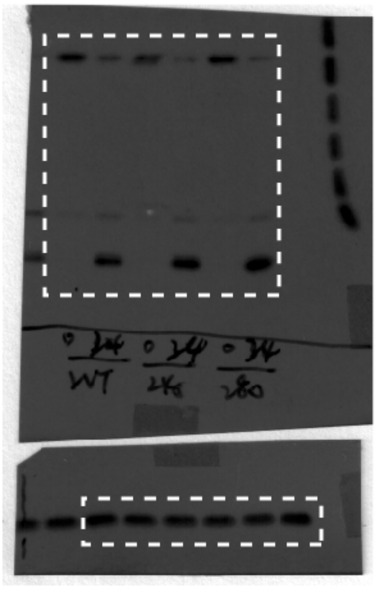

Fig. S3F

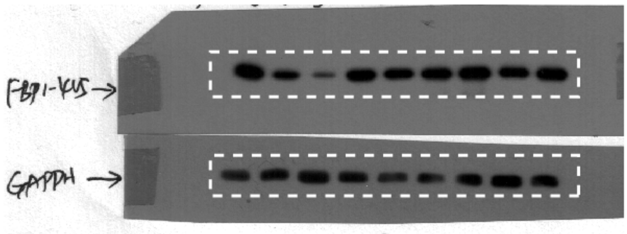

Fig. S4B

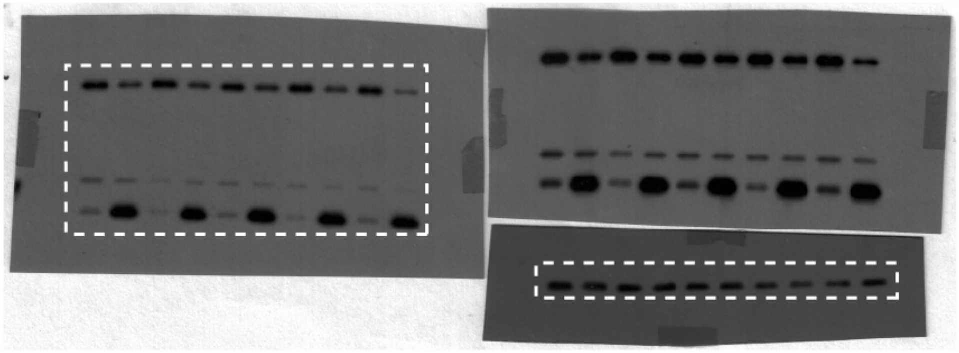

Fig. S4D

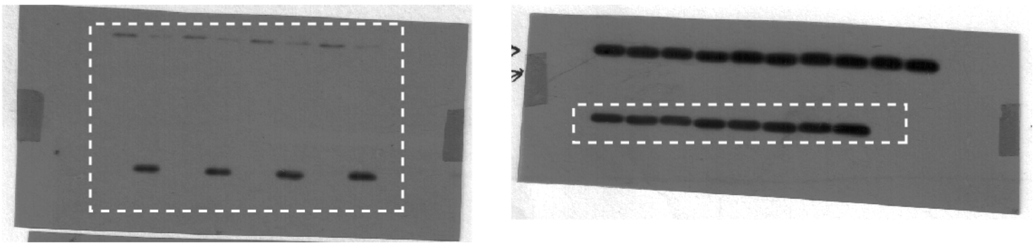

Fig. S6C

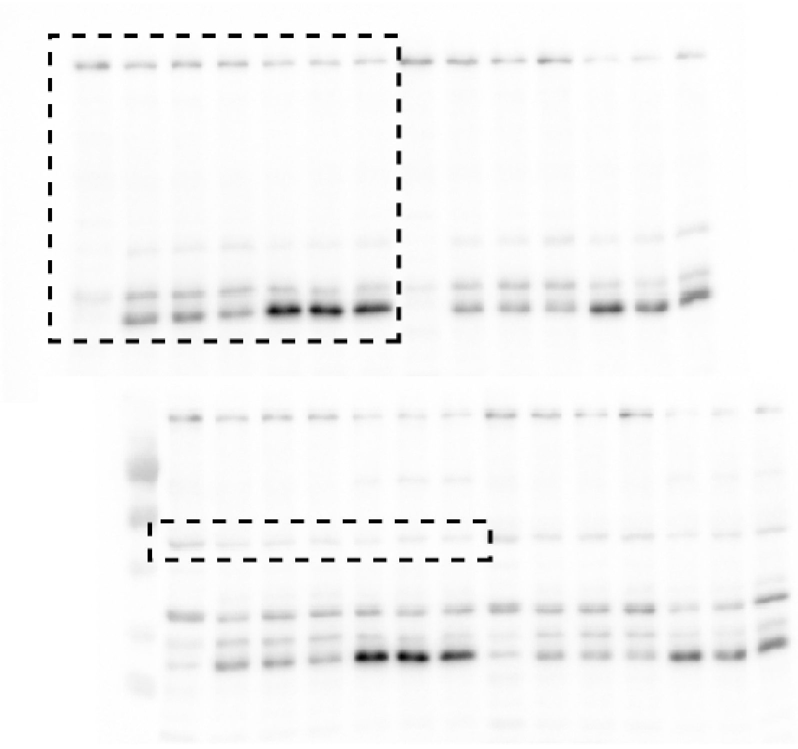

Fig. S7A

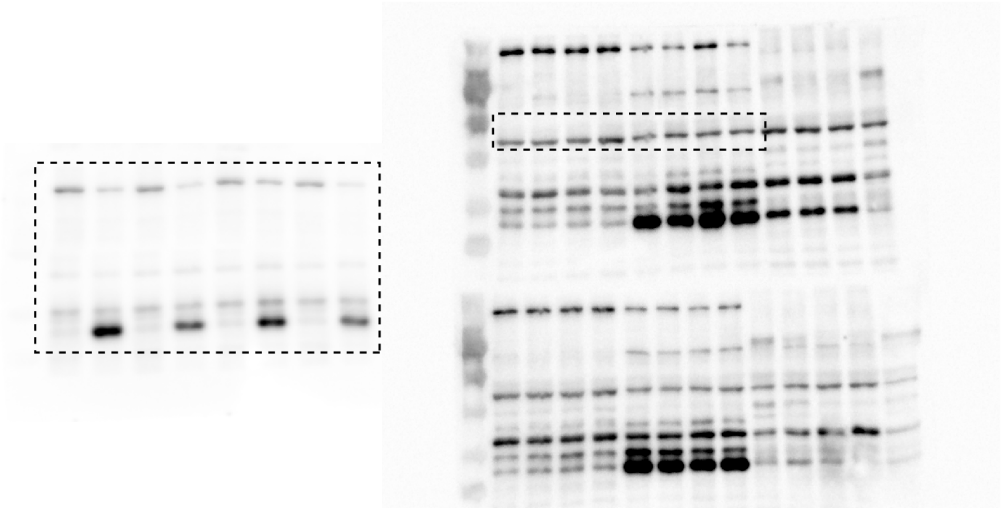

Fig. S7C

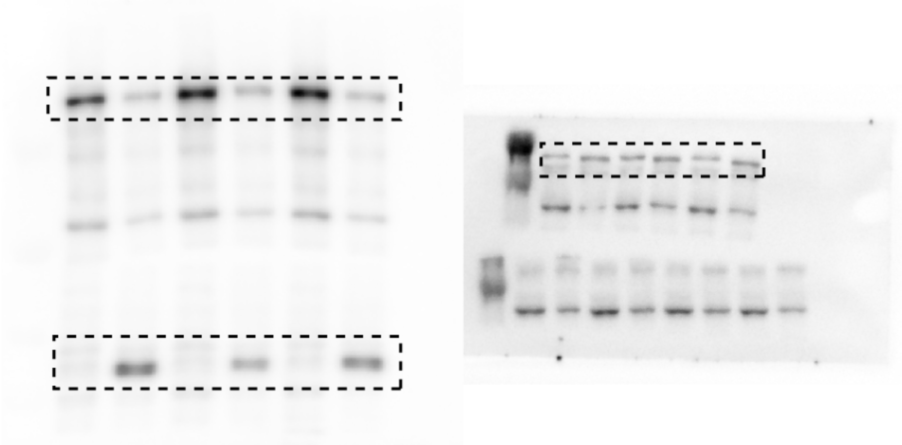

Fig. S8A

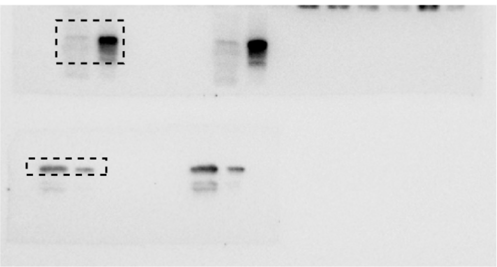

Supplement: Supplementary file 4 — Additional file 4. Uncropped Blots. Uncropped images for all immunoblots in figures and additional file figures. [file 12915_2021_1048_MOESM4_ESM.pdf]
